# VItamin K In PEritonial DIAlysis (VIKIPEDIA): Rationale and study protocol for a randomized controlled trial

**DOI:** 10.1371/journal.pone.0273102

**Published:** 2022-08-17

**Authors:** Stefanos Roumeliotis, Athanasios Roumeliotis, Panagiotis I. Georgianos, Elias Thodis, Leon J. Schurgers, Katarzyna Maresz, Theodoros Eleftheriadis, Evangelia Dounousi, Giovanni Tripepi, Francesca Mallamaci, Vassilios Liakopoulos

**Affiliations:** 1 1st Department of Internal Medicine AHEPA Hospital, Medical School, Aristotle University of Thessaloniki, Thessaloniki, Greece; 2 Department of Nephrology, School of Medicine, Democritus University of Thrace, Alexandroupolis, Greece; 3 Department of Biochemistry, Cardiovascular Research Institute Maastricht, Maastricht, The Netherlands; 4 International Science & Health Foundation, Krakow, Poland; 5 Department of Nephrology, Faculty of Medicine, University of Thessaly, Larissa, Greece; 6 Department of Nephrology, Faculty of Medicine, School of Health Sciences, University of Ioannina, Ioannina, Greece; 7 CNR-IFC, Clinical Epidemiology and Physiopathology of Renal Diseases and Hypertension, Reggio Calabria, Italy; OLV Ziekenhuis Campus Aalst: Onze-Lieve-Vrouwziekenhuis Campus Aalst, BELGIUM

## Abstract

Vascular calcification (VC) is an active process, resulting from the disturbance of balance between inhibitors and promoters of calcification, in favor of the latter. Matrix Gla Protein, a powerful inhibitor of VC, needs vitamin K to become active. In vitamin K depletion, plasma levels of the inactive form of MGP, dephosphorylated, uncarboxylated MGP (dp-ucMGP) are increased and associated with VC and cardiovascular (CV) outcomes. End Stage Renal Disease (ESRD) patients have increased circulating dp-ucMGP levels and accelerated VC. VItamin K In PEritoneal DIAlysis (VIKIPEDIA) is a prospective, randomized, open label, placebo-controlled trial, evaluating the effect of vitamin K2 supplementation on arterial stiffness and CV events in ESRD patients undergoing peritoneal dialysis (PD). Forty-four PD patients will be included in the study. At baseline, dp-ucMGP and pulse-wave velocity (PWV) will be assessed and then patients will be randomized (1:1 ratio) to vitamin K (1000 μg MK-7/day) or placebo for 1.5 years. The primary endpoint of this trial is the change in PWV in the placebo group as compared to the treatment group. Secondary endpoints are the occurrence of CV events, mortality, changes in PD adequacy, change in 24-hour ambulatory blood pressure indexes and aortic systolic blood pressure and changes in calcium/phosphorus/parathormone metabolism. VIKIPEDIA is a new superiority randomized, open label, placebo-controlled trial aiming to determine the effect of vitamin K2 supplementation on VC, CV disease and calcium/phosphorus metabolism, in PD patients.

**Trial registration:** The protocol of this study is registered at ClinicalTrials.gov with identification number NCT04900610 (25 May 2021).

## Introduction

The cardiovascular (CV) burden seen in chronic kidney disease (CKD) patients might be partially explained by the fact that CKD is a state of accelerated vascular calcification (VC) of both the media and intima arterial wall. VC of the arterial tunica media leads to arterial stiffness which is progressively increased in parallel to CKD progression into end- stage kidney disease (ESKD). Pulse wave velocity (PWV), a marker of arterial stiffness, is increased in uremia and is closely associated with CV disease [1]. Compared to hemodialysis (HD), patients undergoing peritoneal dialysis (PD) have higher PWV values and wave reflection indices, suggesting a higher CV risk in this population [2]. Moreover, although several ongoing randomized controlled trials (RCTs) assess various calcification scores as outcomes, we will assess PWV because calcification scores are thought to reflect the final and less dynamic stage of vascular remodeling, whereas PWV might be subjective to change through time [3]. This is why PWV has served as a potential therapeutic target to ameliorate CV risk in ESKD patients in several trials [4].

For a long time, VC was considered as a passive, degenerative process of calcium accumulation within the arterial wall. Our perspective changed during the recent decades, when it was discovered that VC is an active process regulated by inhibitors and promoters. Among these, matrix Gla-protein (MGP) is one of the most powerful natural inhibitors of VC found in the human body. The pivotal clinical role of MGP was first showed in a knock-out experimental rodent model (MGP -/-) that died within 8 weeks from birth due to severe aortic calcification which led to blood-vessel rupture [5]. To become fully active, MGP needs to undergo vitamin K-dependent carboxylation and subsequently serine phosphorylation. Only then MGP can exert its beneficial protective effects against VC [6]. In vitamin K deficiency, high circulating levels of the inactive, dephosphorylated uncarboxylated MGP (dp-ucMGP) are reported in both experimental and clinical studies [7]. Both in vivo and in vitro data suggest that compared to vitamin K1, menaquinone-7 (MK-7), a long-chain isoform of K2, has significantly longer half-life, higher bioavailability and bioactivity [8] and thus we chose MK-7 for our trial.

Both VC and vitamin K deficiency are thought to be interrelated entities that are highly prevalent even at early stages of CKD stages 1+2, are gradually increased along with disease progression to advanced CKD stages 3+4 and are further exacerbated in ESKD (CKD stage 5D) [9]. In CKD and HD populations, dp-ucMGP has been repeatedly associated with various markers of VC and stiffness, including PWV [10,11], whereas accumulating evidence suggest a tight association between circulating dp-ucMGP, mortality and CV disease in pre-dialysis CKD [12–14] and ESKD patients undergoing PD [15]. Of note, of all reported data on dp-ucMGP in CKD populations, only the study by Xu et al., was conducted in PD patients.

In light of the growing body of data supporting the tight association between vitamin K deficiency and VC in uremia, several investigators are currently conducting randomized clinical trials examining the possible therapeutic effect of MK-7 supplementation on VC in ESKD patients [16] undergoing maintenance HD (Trevasc-HDK and Aortic Valve DECalcification trials). In clinicaltrials.gov the search terms “vitamin K” and “hemodialysis” produces 17 results of ongoing or completed RCTs (assessed 24/05/2021). However, none of these trials have been conducted in PD patients, but only in pre-dialysis CKD and/or HD subjects. Additionally, the majority of these trials assessed surrogate VC markers and not clinical hard end-points such as mortality and CV events and no study so far has evaluated the potential effect of MK-7 intake on 24-h ambulatory blood pressure (BP). Further, on-going RCTs in HD patients use daily dosage of MK-7 below 500 μg/day [17]. The precise required MK-7 dosage to restore vitamin K levels to fully activate MGP in ESKD patients is not yet determined. In a dose-finding study in prevalent HD patients, Caluwe et al., showed that a daily dosage of 463 μg MK-7 caused a moderate 46% decrease in dp-ucMGP levels and therefore, it was considered under-therapeutic [18]. Similarly, Westenfeld et al., randomized 53 HD patients, 18 years or older to 3 groups: 45, 135 or 360 μg/day treatment with MK-7 for 6 weeks, with outcome the change in plasma dp-ucMGP levels and found that the response rates in dp-ucMGP reduction were 77% and 93% in the 135 μg and 360 μg group, respectively [19]. Compared to the study by Caluwe, this trial enrolled much younger HD patients who had a better response of 360 μg/day in declining dp-ucMGP, thus suggesting that the therapeutic effect of MK-7 might be dependent on the treated population. Moreover, supplementation of MK-7 dosages <463 μg/day in HD patients failed to show any beneficial effect on VC in a recent RCT [20]. Under these results, in another ongoing RCT in HD patients the proposed dosage of MK-7 is much higher -2 g thrice weekly (trial number NCT04539418). The proposed VItamin K In PEritonial DIAlysis (VIKIPEDIA) study will assess whether high (1mg/day) per os intake of MK-7 can enhance MGP activation, suppress dp-ucMGP and thus improve arterial stiffness and ameliorate CV disease.

Oral administration of vitamin K2 might improve MGP carboxylation status and thus decrease circulating dp-ucMGP. MK-7 is the first and most clinically approved and validated K2 supplement (MenaQ7 ®, Nattopharma, ASA, part of Gnosis by Lesaffre, Lesaffre, France) with proven efficacy in reducing dp-ucMGP [19] and has been used in several clinical trials in HD patients [18,21]. Vitamin K2 is a natural supplement, that can be purchased over the counter from drug stores or even super markets, without physician’s prescription, because it is not a drug. Millions of people around the world are receiving vitamin K2, because it is thought to have several beneficial effects, whereas not toxicity or side effects have been reported. In the VIKIPEDIA study we will administer orally, daily, high dosage of MK-7 to PD patients. Although several clinical trials have administered MK-7 in dosages 200–500 μg/day, this is the first RCT administering 1 mg/day in PD patients. There are no safety concerns with this dosage, because other RCTs have administered MK-7 in ESKD, without reporting thrombotic events, side-effects or complaints [21].

Therefore, since PD patients present significant reduced vitamin K levels compared to pre-dialysis CKD patients, we believe that such a high dose is justified in our cohort and the potential beneficial effects of high-dose treatment might outweigh the potential side-effects. Finally, so far, no study has assessed the pharmacokinetics and pharmacodynamics of dp-ucMGP in PD patients and the concentration of dp-ucMGP in the PD effluent, after a PD session. Therefore, the proposed VIKIPEDIA study is novel and timely.

The major outcome of interest of the VIKIPEDIA study is whether oral administration of MK-7 in PD patients can slow progression of arterial stiffness. We expect that MK-7 supplementation will enhance MGP carboxylation, suppress circulating dp-ucMGP, slower progression of arterial stiffness [assessed by PWV increase] and thus ameliorate CV disease. Further research questions will be whether treatment with MK-7 might reduce all-cause and CV mortality and improve 24-hour ambulatory BP. Moreover, we will assess cross-sectional information regarding the prevalence of arterial stiffness and vitamin K deficiency in PD patients, along with prospective data on the development of arterial stiffness in PD patients in the control group (not treated with MK-7).

## Materials and methods

### Ethics

Our study protocol was developed in accordance with the Helsinki Declaration of Human Rights and the Good Clinical Practice Guidelines and Standard Protocol Items: Recommendations for Intervention Trials [22], was approved by the Ethics Committee/Scientific Council of the Medical School of Aristotle University of Thessaloniki (235/14.05.2021) and is registered at ClinicalTrials.gov with identification number NCT04900610. All participants will provide a structured, written, informed consent.

### Trial design and setting

**Fig 1:**
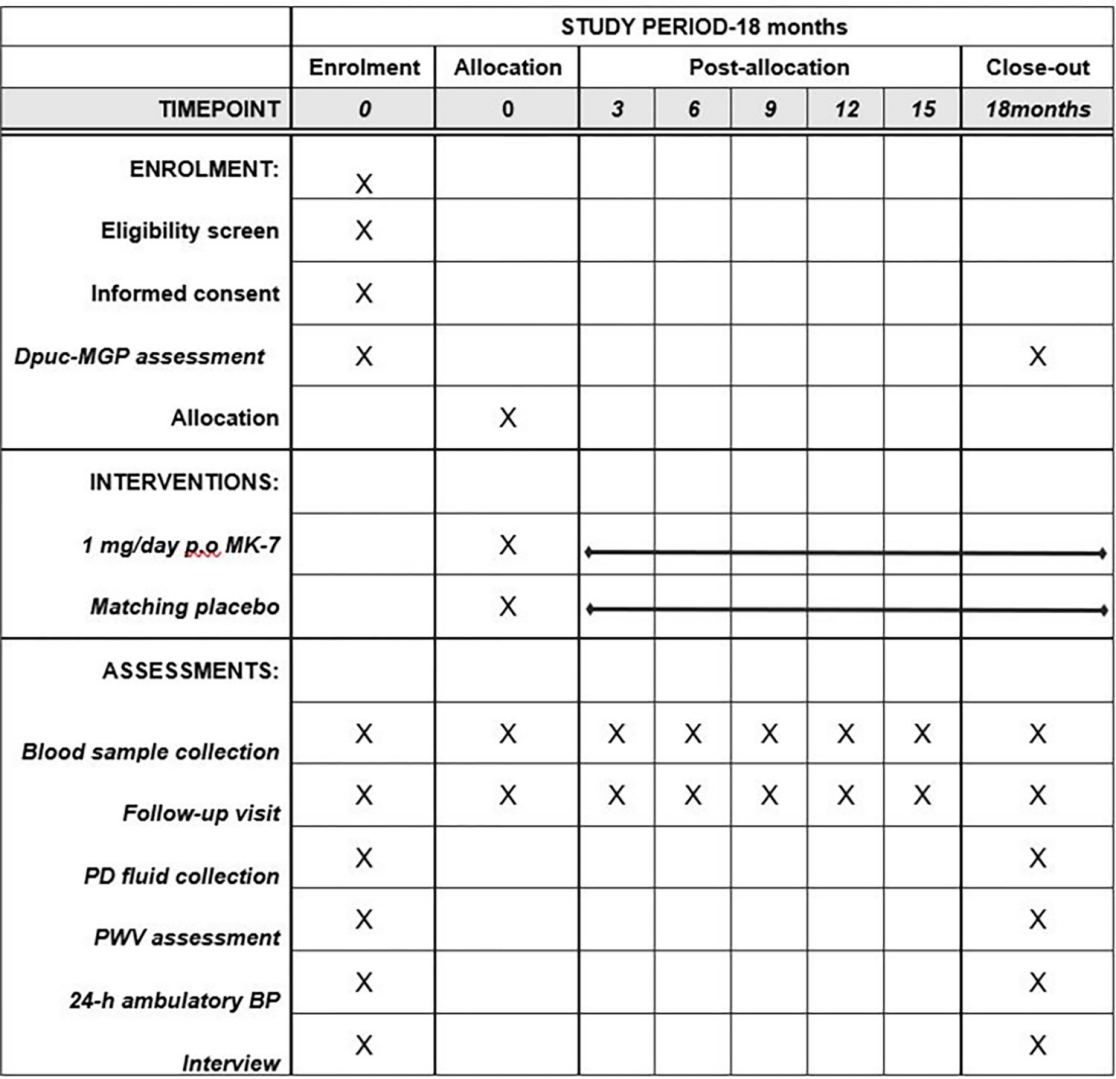
SPIRIT schedule of enrolment, interventions, and assessments of the VIKIPEDIA study.

VIKIPEDIA is a multi-center, placebo-controlled, randomized, open-label intervention clinical trial on PD patients. Three university, tertiary hospitals in Northern Greece with major, referral PD units will participate in the study. The design of the trial is presented in Fig 1. In short, the patients will be recruited within 1 year. At baseline, all eligible patients who have provided a written, informed consent will be enrolled in the study PD and aortic stiffness and vitamin K status will be assessed by PWV and circulating biomarkers dp-ucMGP and PIVKA-II (proteins induced by vitamin K absence factor-II) levels respectively. Before randomization, we will draw blood (serum and plasma) and PD fluid samples from all patients to measure blood count and routine biochemical parameters, including urea, creatinine, potassium, sodium, calcium, phosphorus, c-reactive protein, alkaline phosphatase, albumin, parathormone, 25-OH D3, magnesium, glycated hemoglobin, thyroid function hormones. Since both vitamin D and magnesium are considered of utmost importance in calcification, after baseline, patients with vitamin D and/or magnesium depletion will be treated with oral supplements to achieve normal levels of both elements, before randomization. We will aim to maintain the serum levels of magnesium between 1.3 and 2.1 mEq/L (0.65–1.05 mmol/L), as described before [23], and the target serum 25 (OH) D levels will be above 30 mg/ml, as reccomended by the Kidney Disease Outcomes Quality Initiative [24] and Kidney Disease Improving Global Outcomes guidelines [25]. Our cohort will then be categorized to one of the two groups (placebo or active group) and the treatment period will last 1.5 years. The design and flow of the VIKIPEDIA study is shown in **Fig 1**. As mentioned before, vitamin K2 is a natural supplement, that can be purchased over the counter and it is not a drug.

To ensure that the two parallel groups will include patients that will not differ significantly in vitamin K and stiffness we will stratify the patients accordingly. However, since the clinicians that will assess PWV, 24hour BP and the study endpoints will be blinded to the treatment, information bias is excluded. After randomization, all patients will continue their routine, standard medical treatment and patients in the treatment group will additionally receive daily, per os 1 mg of vitamin K2 (MenaQ7 ®, Nattopharma, part of Gnosis by Lesaffre, Lesaffre, France).

### Inclusion and exclusion criteria

Inclusion and exclusion criteria of this study are shown in Table 1. Since sevelamer has been reported to reduce the bioavailability of vitamin K, we will discontinue the use of this agent in patients that fulfill all inclusion criteria and start another calcium/ or iron-based phosphate binder. After a wash-out period of 21 days, patients will be enrolled in the study. Since this is a study only including PD patients, in case a patient will be transferred to HD automatically he/she is excluded from the analysis.

**Table 1:** Inclusion and exclusion criteria of the VIKIPEDIA study.

Inclusion criteria	Exclusion criteria
Age ≥ 18 years	Liver disease
At least 3 months on PD	Drug or alcohol abuse
Life expectancy of ≥ 18 months	Pregnancy or breast-feeding
	Ongoing malignancy or severe inflammatory disease diagnosis
	Use of vitamin K antagonist or vitamin K supplements during the past 3 months
	Diagnosis of severe gut-disease (inflammatory or short bowel disease) or gastrointestinal malabsorption
	Mental disorder rendering the patient unable to conform with the instructions and fully understand the nature, aim and possible side-effects of the supplementation

### Marker of vitamin K deficiency

As a marker of vitamin K deficiency, we will assess plasma dp-ucMGP at baseline and the end of the study. Additional parameters that will be measured are vitamin K plasma concentration and PIVKA-II. Blood will be obtained from patients and plasma will be immediately stored at -80°C, until dry ice transfer for analysis at Coagulation Profile, Maastricht, the Netherlands [26]. Circulating dp-ucMGP will be assessed using a sandwich enzyme linked immunosorbent assay (ELISA) based on two anti-MGP monoclonal antibodies, directed against the uncarboxylated MGP domain 35–49 (mAb-ucMGP; VitaK BV, Maastricht, The Netherlands) and against the non-phosphorylated MGP domain 3–15 (mAb-dp-MGP; VitaK BV), as described before [13,27]. Circulating PIVKA-II levels will be measured in plasma using a conformation-specific monoclonal antibody in a commercially available competitive ELISA assay as described before [28,29]. Vitamin K levels will be measured by HPLC separation (using a C-18 reversed phase column) and fluorescence detection after post-column electrochemical decrease as described elsewhere [30].

### Marker of arterial stiffness

One of the primary outcomes of the trial is the increase in arterial stiffness, evaluated by PWV.

### Tonometric measurement of arterial stiffness and central aortic BP with the Sphygmocor device

Radial artery applanation tonometry with a high-fidelity, pencil-type SPT-301 (Millar Instruments, Houston, TX) probe interfaced with a computer running Sphygmocor software (ArtCor, Sydney, Australia), will be performed to estimate central hemodynamic indices. The Sphygmocor software regenerates the aortic pulse waveform via mathematical transformation of the radial pulse waveform (generalized transfer function). Aortic pulse waveform will be calibrated by inserting the brachial systolic BP (bSBP) and brachial diastolic BP (bDBP) recorded immediately before the Sphygmocor measurement [31]. Augmentation pressure (AP) will be defined as the difference of aortic pressures between the second and first systolic peaks. Augmentation index (AIx) will be calculated as the ratio of AP to aortic pulse pressure (PP) and will be expressed as percentage (%). Heart rate-adjusted AIx (AIx(75)) will be estimated by adjusting AIx at an inverse rate of 4.8% for each 10 beats per minute increase in heart rate (2;6). Aortic PWV will be determined by performing applanation tonometry at the carotid and femoral arteries with the above-described pencil-type tonometer [32]. Pulse waveforms will be referenced to a concurrently recorded ECG, and pulse wave transit time between the subsequent recording sites will be calculated using the foot-to-foot time difference between carotid/femoral waveforms [33]. Body surface distances from the suprasternal notch to the carotid recording site (distance A) and from the suprasternal notch to the femoral recording site (distance B) will be measured and pulse wave travel distance will be calculated by subtracting the distance B from distance A. Aortic PWV will be estimated by dividing the pulse wave travel distance to transit time. We will measure PWV over ten consecutive heartbeats to cover a complete respiratory cycle. The first valid tonometric measurement will be used in statistical analysis [31].

Secondary outcomes will be the changes in ambulatory BP indices.

The tonometric measurements will be performed in a quiet room with stable temperature (21 oC) after an at least 5 minutes rest period in the supine position by a single, well-trained physician. The patients will be also instructed to refrain from smoking and caffeine consumption at least 1 hour prior to the study evaluations.

### ABPM with the Mobil-O-Graph device

Brachial and central aortic BP, AIx and PWV will be recorded under ambulatory conditions over 24 hours with the brachial cuff-based oscillometric device Mobil-O-Graph (IEM, Stolberg, Germany) [34]. The BP-detection unit of this device was validated according to the criteria of European Society of Hypertension/European Society of Cardiology (ESH/ESC). The monitor is programmed to measure BP 3 times per hour during daytime (07:00 to 22:59) and 2 times per hour during nighttime (23:00 to 06:59). ABPM will be considered complete when >80% of readings will be valid with ≤2 non-consecutive daytime hours with <2 valid recordings and ≤1 nighttime hour without valid recording. Participants with incomplete or invalid recordings will be asked to repeat ABPM within the next week.

The methodology incorporated by the Mobil-O-Graph device is described in detail elsewhere [35]. After the oscillometric recording of brachial BP, the cuff re-inflates at the diastolic phase, acquiring the brachial pressure waveforms for ~10 seconds with a high-fidelity pressure sensor (MPX5050, Freescale, Tempe, AZ, USA). Subsequently, the software (HMS version 4.5) regenerates the aortic pulse waveform by means of an ARCSolver algorithm using a generalized transfer function. The calculation of central aortic pressures was based on the C1 (brachial systolic BP /diastolic BP) calibration method. The Mobil-O-Graph device performs also wave separation analysis by decomposing the aortic pulse waveform into forward- and backward-traveling pulse waves with a triangular aortic flow waveform [33]. Utilizing parameters from pulse wave and wave separation analyses, the ARCSolver algorithm estimates the following indices:

- augmentation pressure, defined as the difference of the pressure at second minus the pressure at first inflection point of the systolic phase of pulse wave;
- AIx, an index of pulse wave reflection at the level of microcirculation, defined as the ratio of augmentation pressure to aortic PP;
- PWV, a direct marker of arterial stiffness, calculated from the reconstructed aortic pulse waveform via mathematical algorithms, taking into account the characteristic impedance and age and assuming a three-element Windkessel model.

### Outcomes

We will consider the primary endpoint of aortic stiffness’ progression, assessed by the absolute change in the value of PWV after 1.5 years of treatment compared to PWV at baseline before the initiation of treatment.

We will consider the following secondary endpoints:

- The occurrence of non-fatal CV events, including acute myocardial infarction, acute coronary syndrome, embolism, peripheral arterial disease and stroke.
- The occurrence of all-cause and CV mortality

- The absolute change from baseline in the values of PWV indices, wave reflection indices, heart-rate-adjusted augmentation index, SVRI and PP
- The percentage change over time in the values of PWV indices, wave reflection indices, heart-rate-adjusted augmentation index SVRI and PP
- PD adequacy (RRF preservation, Kt/V)
- Rate of infections and peritonitis
- The absolute change in 24-hour ambulatory BP indexes and aortic systolic BP
- Changes in serum parathormone from baseline
- Changes in the calcium phosphorus product from baseline
- Fracture incidence
- Incidence of joint/muscle pain

### Statistical analysis

All eligible patients who provided informed consent will be enrolled and randomized to the treatment or placebo group (on a 1:1 ratio). The primary endpoint is progression of arterial stiffness, defined as the absolute change in PWV value at the end of the 18 months versus the baseline. We will measure the change in PWV in the control arm and in the treatment arm at baseline (0) and at the end of the study (18 months) and we will compare the difference between changes of the two arms by T-test or Mann-Whitney test, as appropriate. The change of PWV in each arm will be expressed as mean and the 95% confidence interval of the mean difference. Our analysis will be primarily conducted by the intention-to-treat approach. A per protocol analysis will be also performed. If necessary, appropriate statistical methods will be applied to account for potential confounders. Primary and secondary outcomes will be assessed as difference between arms at 18 months by two-way analysis of covariance (ANCOVA). Since age and diabetes might significantly alter the treatment effect, we will perform a prespecified subgroup analysis in patients with and without diabetes and patients over versus under 65 years. We will conduct multiple imputation for all missing data, using the average of five imputed data sets for the outcomes [36]. Regarding the safety population, we will include in the analysis only patients that will actually receive either MK-7 or placebo. These patients will be grouped for analysis according to the treatment that they actually received, as opposed to the treatment that they were categorized to receive at baseline-randomization stage. The Statistical analysis plan for the final analysis will be finalized and approved before the data lock for the final analysis.

### Sample size calculation

This is a superiority trial, assessing a continuous outcome as the primary endpoint. Until to-date no RCTs have been conducted in PD patients, therefore our calculation will be based on already published data in similar populations. We calculated the sample size for the primary outcome of arterial stiffness progression (continuous outcome), based on previous studies [37,38]. The Renakvit RCT [3], is a recently published study, with similar design and population. This study was designed to give an 80% power to detect 1 m/s difference in PWV after 1 year at a significance level of 5% at 0.95 m/s assumed SD [37,38]. Expecting a yearly drop-out rate of 20%, this study aimed at enrolling 40 patients (2x20 per group). Based on these studies, we expect that at the end of the study, the increase in PWV value (absolute difference from baseline to the end of the study) will be 1 m/s higher in the placebo group than in the treatment group with an assumed standard deviation of 0.95 m/s [3,37,38]. Accounting for an estimating drop-out rate of 30%, with a two-sided significance level of 5% and 80% power, 44 patients will be required (22 in each group) to detect the significant treatment difference.

### Randomization

This is a superiority, open label, placebo-controlled RCT. The randomization will be central and simple, performed by using the Random Allocation Software. The allocation concealment will be guaranteed, because the randomization list will be centrally maintained and participating centers will receive the allocation arm once they will communicate to the randomization center the patients’ identification. The study will be open label. To avoid assessment bias, the clinicians that will assess PWV, 24hour BP and the study endpoints will be blinded to the treatment (PROBE approach). No stratification will be adopted.

### Monitoring-safety considerations

Next to baseline, during the study period, 6 follow-up visits will take place at months 3, 6, 9, 12, 15 and 18 respectively. During all these visits, patients will be interviewed regarding the compliance and potential side effects (and pills will be counted), and clinical examination and routine blood sampling will be performed. To evaluate the patients’ compliance, the drug boxes will be thoroughly checked and remaining pills will be counted and reported. As before enrollment, all patients will undergo standard, regular follow-up visits every month in their PD unit. At the end of the follow-up period, all plasma and serum parameters that were measured at baseline, along with PWV will be re-assessed. The occurrence of the trial’s endpoints will be documented by death certificates, medical files and records and integrated interview at the last visit or telephone interviews. During the study period, all potential adverse events will be closely monitored and recorded. These data will be evaluated thoroughly.

### Status

Participant recruitment and data collection has not started yet.

### Discussion

In this paper, we present the rationale and study protocol for the prospective, randomized, placebo-controlled VIKIPEDIA trial. To our knowledge this is the first intervention with vitamin K supplementation in PD patients. There is a growing body of evidence suggesting that the vast majority of ESKD patients suffer from vitamin K deficiency and carry a heavy CV burden. Currently, there are several RCTs investigating the possible beneficial effect of vitamin K supplementation on ESKD patients undergoing maintenance HD. This is the first trial aiming to evaluate the effect of vitamin K2 supplementation on arterial stiffness and CV events in ESKD patients undergoing PD.

The open-label design of the study might be considered as a limitation of this study. However, this was necessary to ensure that patients would be divided in groups according to their vitamin K status and thus we would be able to investigate the possible beneficial clinical effects of vitamin K supplementation on patients with vitamin K deficiency. The criteria for early termination of the trial will be withdraw of the written consent, death, kidney transplantation, transfer to HD, severe allergic response to MK-7 and medical necessity for treatment initiation with vitamin K antagonists. Our study results will be disseminated through international journals. We will inform all patients and participants’ support groups about the results.

The VIKIPEDIA study is the first intervention study investigating the effect of MK-7 supplementation on various hard CV endpoints on PD patients. Since the management of the heavy CV burden of PD patient is critical, we expect that this study will provide answers regarding the possible beneficial effect of MK-7 supplementation in these patients.

## Supporting information

S1 File SPIRIT checklist. (DOCX)

S2 File Submitted protocol proposal in ethics committee and consent form in English. (DOCX)

S3 File Submitted protocol proposal in ethics committee and consent form in Greek. (DOCX)
