# Supplementary material for: VItamin K In PEritonial DIAlysis (VIKIPEDIA): Rationale and study protocol for a randomized controlled trial
Source: PLoS One. 2022 Aug 17;17(8):e0273102. doi: 10.1371/journal.pone.0273102 (PMC9384975; doi:10.1371/journal.pone.0273102)
Supplement: S2 File — (DOCX) [file pone.0273102.s002.docx]

Ethics Committee/Scientific Council

Secretary: V. Kalampoka

Tel: 2313303709

**Thessaloniki, 14.05.2021**

Identification number: 235

Subject 49^th^: The application with identification number 9322/12.03.2021 of Ass. Professor of Nephrology V. Liakopoulos and Chief of the First Department of Internal Medicine of AHEPA Hospital, Professor P. Zebekakis are submitting the protocol “Vitamin K In PEritonial DIAlysis (VIKIPEDIA)” for a research study that will be conducted with Ass. Professor V. Liakopoulos as Scientific Supervisor.

**The Research Ethics Committee/Scientific Council at the 10^th^/12.05.2021 meeting examined:**

1. The application with identification number 9322/12.03.2021 of Ass. Professor of Nephrology V. Liakopoulos and Chief of the First Department of Internal Medicine of AHEPA Hospital, Professor P. Zebekakis asking approval for the research study “Vitamin K In PEritonial DIAlysis (VIKIPEDIA): Rationale and study protocol for a randomized controlled trial” that will be conducted with Ass. Professor V. Liakopoulos as Scientific Supervisor. In the detailed protocol it is clearly reported that conducting this study does not require any funding by the Hospital. All information and data that will be collected will remain confidential and coded. Participation will be voluntary and only after providing a written, informed consent.
2. The study research protocol and the current literature
3. The informed consent form for participation in the study
4. The main purpose of the study, which is if per os supplementation with MK-7 in peritoneal dialysis patients might delay progression of arterial stiffness
5. The discussion of all members of the board

**And reached the following unanimous decision:**

**We approve the conducting of the randomized controlled study** “Vitamin K In PEritonial DIAlysis (VIKIPEDIA)” with Ass. Professor V. Liakopoulos as Scientific Supervisor in the First Department of Internal Medicine of AHEPA Hospital. Any economic burden that might arise will not be covered by the hospital, the participation in the study will be voluntary and only after providing a written, informed consent, all information and data that will be collected will remain confidential and coded and after the end of the study a copy of the results of the study should be submitted in the Medical Library of the Hospital.

The President of the Ethics Committee/Scientific Council


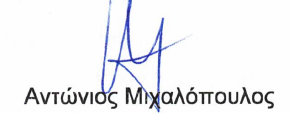


Professor of Surgery, Aristotle University of Thessaloniki

**Abstract**

Vascular calcification (VC) is an active process, resulting from the disturbance of balance between inhibitors and promoters of calcification, in favor of the latter. Matrix Gla Protein, a powerful inhibitor of VC, needs vitamin K to become active. In vitamin K depletion, plasma levels of the inactive form of MGP, dephosphorylated, uncarboxylated MGP (dp-ucMGP) are increased and associated with VC and cardiovascular (CV) outcomes. End Stage Renal Disease (ESRD) patients have increased circulating dp-ucMGP levels and accelerated VC. VItamin K In PEritoneal DIAlysis (VIKIPEDIA) is a prospective, randomized, placebo-controlled trial, evaluating the effect of vitamin K2 supplementation on arterial stiffness and CV events in ESRD patients undergoing peritoneal dialysis (PD). One hundred and twenty PD patients will be included in the study. At baseline, dp-ucMGP and pulse-wave velocity (PWV) will be assessed and then patients will be randomized (1:1 ratio) to vitamin K (1000 μg MK-7/day) or placebo for 1.5 years. The primary endpoint of this trial is the change in PWV in the placebo group as compared to the treatment group and the occurrence of CV events. Secondary endpoints are mortality, changes in PD adequacy, change in 24-hour ambulatory blood pressure indexes and aortic systolic blood pressure and changes in calcium/phosphorus/parathormone metabolism.

VIKIPEDIA is a new trial aiming to determine the effect of vitamin K2 supplementation on VC, CV disease and calcium/phosphorus metabolism, in PD patients. The protocol of this study is registered at ClinicalTrials.gov with identification number NCT04900610.

1. **Introduction**

The heavy cardiovascular (CV) burden seen in these patients might be partially explained by the fact that chronic kidney disease (CKD) is a state of accelerated vascular calcification (VC) of both the media and intima arterial wall. VC of the arterial tunica media leads to arterial stiffness which is progressively increased in parallel to CKD progression to End-Stage Kidney Disease (ESKD). Pulse wave velocity (PWV), a marker of arterial stiffness, is increased in uremia and is closely associated with CV disease (1). Compared to hemodialysis (HD), patients undergoing Peritoneal Dialysis (PD) have higher PWV values and wave reflection indices, suggesting a possible higher CV risk in this population (2). Moreover, although several ongoing randomized controlled trials (RCTs) assess various calcification scores as outcomes, we will chose PWV because calcification scores are thought to reflect the final and less dynamic stage of VC, whereas PWV might be subjective to change through time (3). This is why, PWV has served as a potential therapeutic target to ameliorate CV risk in ESKD patients in several trials (4).

For a long time, VC was considered as a passive, degenerative process of calcium accumulation within the arterial wall. Our perspective changed during the recent decades, when it was discovered that VC is an active process regulated by inhibitors and promoters. Among these, Matrix Gla Protein (MGP) is the most powerful natural inhibitor of VC found in the human body. The pivotal clinical role of MGP was firstly showed in knock-out experimental models (MGP -/-) that died within 8 weeks from birth due to severe aortic calcification which led to blood-vessel rupture (5). To become fully active, MGP needs to undergo vitamin K2-dependent carboxylation and then vitamin K2-dependent phosphorylation. Only then MGP can exert its beneficial protective effects against VC (6). In vitamin K deficiency, high circulating levels of the inactive, dephosphorylated uncarboxylated MGP (dp-ucMGP) are reported in both experimental and clinical studies (7). Both in vivo and in vitro data suggest that compared to vitamin K1, menaquinone-7 (MK-7), a long-chain isoform of K2, has significantly longer half-life, higher bioavailability and bioactivity (8) and thus its supplementation is preferred over K1 in RCTs.

Both VC and vitamin K deficiency are thought to be interrelated entities that are highly prevalent even at early stages of Chronic Kidney Disease (CKD) stages 1+2, are gradually increased along with disease progression to advanced CKD stages 3+4 and are further exacerbated in ESKD (CKD stage 5) (9). In CKD and HD populations, dpuc-MGP has been repeatedly associated with various markers of VC and stiffness, including PWV (10, 11), whereas accumulating evidence suggest a tight association between circulating dp-ucMGP, mortality and CV disease in pre-dialysis CKD (12-14) and ESKD patients undergoing maintenance HD (15) or PD (16). Of note, of all the reported data on dp-ucMGP in CKD populations, only the study by Xu et al., was conducted in PD patients.

In the light of the growing body of data supporting the tight association between vitamin K deficiency and VC in uremia, several investigators are currently conducting randomized clinical trials examining the possible therapeutic effect of MK-7 supplementation on VC in ESKD patients (17) undergoing maintenance HD (BASIK2, VitaVasK, VitaK-CAC, Trevasc-HDK and Aortic Valve DECalcification trials). In *clinicaltrials.gov* the search terms “vitamin K” and “hemodialysis” produces 20 results of ongoing or completed RCTs (assessed 23/02/2021). However, none of these trials have been conducted in PD patients, but only in pre-dialysis CKD and/or HD subjects. Moreover, the majority of these trials assessed surrogate VC markers and not clinical hard end-points such as mortality and CV events and no study so far has evaluated the potential effect of MK-7 intake on 24-h ambulatory blood pressure (BP). Another issue of on-going RCTs in HD patients is that the daily dosage of MK-7 is usually below 500 μg/day (18). The precise required MK-7 dosage to restore vitamin K depletion and thus fully activate MGP in ESKD patients is not yet determined. However, in a dose-finding study in prevalent HD patients, Caluwe et al., showed that a daily dosage of 463 μg MK-7 caused a moderate 46% decrease in dp-ucMGP levels and therefore, it was considered under-therapeutic (19). Moreover, supplementation of MK-7 dosages<463 μg/day in HD patients failed to show any beneficial effect on VC in a recent RCT (20). Under these results, in another ongoing RCT in HD patients the proposed dosage of MK-7 is much higher -2 g thrice weekly (trial number *NCT04539418).* The proposed Vitamin K In PEritonial DIAlysis (VIKIPEDIA) study will assess whether high (1g/day) per os intake of MK-7 can enhance MGP activation, suppress dp-ucMGP and thus improve arterial stiffness and ameliorate CV disease.

Oral administration of vitamin K2 might improve MGP carboxylation and phosphorylation status and thus decrease circulating dp-ucMGP. Vitamin K2 (Menaquinone-7, MK-7) is the first and most clinically approved and validated K2 supplement (MenaQ7 ®, Nattopharma, ASA, Hovik, Norway) with proven efficacy in reducing dp-ucMGP (21) and has been used in several clinical trials in HD patients (19, 22). Vitamin K2 is a natural supplement, that can be purchased over the counter from drug stores or even super markets, without physician’s prescription, because it is not a drug. Millions of people around the world are receiving vitamin K2, because it is thought to have several beneficial effects, whereas not toxicity or side effects have been reported. In the VIKIPEDIA study we will administer orally, daily, high dosage of MK-7 to PD patients. Although several clinical trials have administered MK-7 in dosages 200-500 μg/day, this is the first RCT administering 1g/day in PD patients. There are no safety concerns with this dosage, because other RCTs have administered MK-7 in ESKD, without reporting thrombotic events, side-effects or complaints (22).

Therefore, since ESKD patients undergoing PD present significant reduced K2 levels compared to pre-dialysis CKD patients, we believe that such a high dose is justified in our cohort and the potential beneficial effects of high-dose treatment might outweigh the potential side-effects. Finally, so far, no study has assessed the pharmacokinetics and pharmacodynamics of dp-ucMGP in PD patients and the concentration of dp-ucMGP in the PD effluent, after a PD session. Therefore, the proposed VIKIPEDIA study is novel and timely.

1. **Objectives**

The major outcome of interest of the VIKIPEDIA study is whether oral administration of MK-7 in ESKD patients undergoing PD can slow progression of arterial stiffness. We expect that MK-7 supplementation will enhance MGP carboxylation and phosphorylation, suppress circulating dp-ucMGP, slower progression of arterial stiffness [assessed by PWV increase] and thus ameliorate CV disease. Further research questions will be whether treatment with MK-7 might reduce all-cause and CV mortality and improve 24-hour ambulatory BP. Our research hypothesis is that daily, high dosage of MK-7 supplementation might increase γ-carboxylation and thus activation of MGP, decrease circulating dp-ucMGP and thus ameliorate CV disease in the PD population. Moreover, we will assess cross-sectional information regarding the prevalence of arterial stiffness and vitamin K deficiency in PD patients, along with prospective data on the development of arterial stiffness in PD patients in the control group (not treated with MK-7).

1. **Materials and Methods**

*Trial Design and Setting*

VIKIPEDIA is a multi-centre, placebo-controlled, randomized, open-label intervention clinical trial on PD patients. Our study protocol was developed in accordance with the Helsinki Declaration of Human Rights and the Good Clinical Practice Guidelines and Standard Protocol Items: Recommendations for Intervention Trials (23), was approved by the Ethics Committee/Scientific Council of the Medical School of Aristotle University of Thessaloniki (235/14.05.2021). All participants will provide a structured, written, informed consent. Three university, tertiary hospitals in Northern Greece with major, referral PD units will participate in the study. The design of the trial is presented in figure 1. In short, the patients will be recruited within 1 year. At baseline, all eligible patients who have provided a written, informed consent will be enrolled in the study. Αortic stiffness and vitamin K status will be assessed by PWV and plasma dp-ucMGP levels respectively. Before randomization, we will draw blood (serum and plasma) and PD fluid samples from all patients to measure blood count and routine biochemical parameters, including urea, creatinine, potassium, sodium, calcium, phosphorus, c-reactive protein, alkaline phosphatase, albumin, parathormone, 25-OH D3, magnesium, glycated hemoglobin, thyroid function hormones. Since both vitamin D and magnesium are considered of utmost importance in vitamin K metabolism, after baseline, patients with vitamin D and/or magnesium depletion will be treated with oral supplements to achieve normal levels of both elements, before randomization. Our cohort will then be categorized to one of the two groups (placebo or active group) and the treatment period will last 1.5 years. As mentioned before, vitamin K2 is a natural supplement, that can be purchased over the counter and it is not a drug.

To ensure that the two parallel groups will include patients that will not differ significantly in vitamin K and stiffness we will stratify the patients accordingly. However, since the clinicians that will assess PWV, 24hour BP and the study endpoints will be blinded to the treatment, information bias is excluded. After randomization, all patients will continue their routine, standard medical treatment and patients in the treatment group will additionally receive daily, per os 1 g of vitamin K2 (MenaQ7 ®, Nattopharma, ASA, Hovik, Norway).

*Inclusion and exclusion criteria*

Inclusion and exclusion criteria of this study are shown in Table 1.

*Marker of vitamin K deficiency*

As a marker of vitamin K deficiency we will assess plasma dp-ucMGP at baseline and the end of the study. Additional parameters that will be measured are vitamin K plasma concentration and proteins induced by vitamin K absence-II (PIVKA-II). Blood will be obtained from patients and plasma will be immediately stored at -80°C, until dry ice transfer for analysis in VitaK, Maastricht, the Netherlands, as described before (24).

*Measurement of arterial stiffness*

One of the primary outcomes of the trial is the increase in arterial stiffness, evaluated by PWV.

*Tonometric measurement of arterial stiffness and central aortic BP with the Sphygmocor device*

Radial artery applanation tonometry with the a high-fidelity, pencil-type SPT-301 (Millar Instruments, Houston, TX) probe interfaced with a computer running Sphygmocor software (ArtCor, Sydney, Australia), will be performed to estimate central hemodynamic indices. The Sphygmocor software regenerates the aortic pulse waveform via mathematical transformation of the radial pulse waveform (generalized transfer function). Aortic pulse waveform will be calibrated by inserting the brachial systolic BP (bSBP) and brachial diastolic BP (bDBP) recorded immediately before the Sphygmocor measurement (25). Augmentation pressure (AP) will be defined as the difference of aortic pressures between the second and first systolic peaks. Augmentation index (AIx) will be calculated as the ratio of AP to aortic pulse pressure (PP) and will be expressed as percentage (%). Heart rate-adjusted AIx (AIx(75)) will be estimated by adjusting AIx at an inverse rate of 4.8% for each 10 beats per minute increase in heart rate (2;6). Aortic PWV will be determined by performing applanation tonometry at the carotid and femoral arteries with the above-described pencil-type tonometer (26). Pulse waveforms will be referenced to a concurrently recorded ECG, and pulse wave transit time between the subsequent recording sites will be calculated using the foot-to-foot time difference between carotid/femoral waveforms (27). Body surface distances from the suprasternal notch to the carotid recording site (distance A) and from the suprasternal notch to the femoral recording site (distance B) will be measured and pulse wave travel distance will be calculated by subtracting the distance B from distance A. Aortic PWV will be estimated by dividing the pulse wave travel distance to transit time. We will measure PWV over ten consecutive heartbeats to cover a complete respiratory cycle. The first valid tonometric measurement will be used in statistical analysis (25).

Secondary outcomes will be the changes in ambulatory BP indices.

*ABPM with the Mobil-O-Graph device*

Brachial and central aortic BP, AIx and PWV will be recorded under ambulatory conditions over 24 hours with the brachial cuff-based oscillometric device Mobil-O-Graph (IEM, Stolberg, Germany) (28). The BP-detection unit of this device was validated according to the criteria of European Society of Hypertension/European Society of Cardiology (ESH/ESC). The monitor is programmed to measure BP 3 times per hour during daytime (07:00 to 22:59) and 2 times per hour during nighttime (23:00 to 06:59). ABPM will be considered complete when >80% of readings will be valid with ≤2 non-consecutive daytime hours with <2 valid recordings and ≤1 nighttime hour without valid recording. Participants with incomplete or invalid recordings will be asked to repeat ABPM within the next week.

The methodology incorporated by the Mobil-O-Graph device is described in detail elsewhere (29). After the oscillometric recording of brachial BP, the cuff re-inflates at the diastolic phase, acquiring the brachial pressure waveforms for ~10 seconds with a high-fidelity pressure sensor (MPX5050, Freescale, Tempe, AZ, USA). Subsequently, the software (HMS version 4.5) regenerates the aortic pulse waveform by means of an ARCSolver algorithm using a generalized transfer function. The calculation of central aortic pressures was based on the C1 (brachial systolic BP /diastolic BP) calibration method. The Mobil-O-Graph device performs also wave separation analysis by decomposing the aortic pulse waveform into forward- and backward-traveling pulse waves with a triangular aortic flow waveform (27). Utilizing parameters from pulse wave and wave separation analyses, the ARCSolver algorithm estimates the following indices:

1. augmentation pressure, defined as the difference of the pressure at second minus the pressure at first inflection point of the systolic phase of pulse wave;
2. AIx, an index of pulse wave reflection at the level of microcirculation, defined as the ratio of augmentation pressure to aortic PP;
3. PWV, a direct marker of arterial stiffness, calculated from the reconstructed aortic pulse waveform via mathematical algorithms, taking into account the characteristic impedance and age and assuming a three-element Windkessel model.

*Study endpoints*

We will consider two primary endpoints:

-Progression of aortic stiffness, assessed by the absolute change in the value of PWV after 1.5 years of treatment compared to PWV at baseline before the initiation of treatment.

-The occurrence of non-fatal CV events, including acute myocardial infarction, acute coronary syndrome, embolism, peripheral arterial disease and stroke.

We will consider the following secondary endpoints:

-All-cause and CV mortality

- The absolute change from baseline in the values of PWV indices, wave reflection indices, heart-rate-adjusted augmentation index, SVRI and PP

-The percentage change over time in the values of PWV indices, wave reflection indices, heart-rate-adjusted augmentation index SVRI and PP

-PD adequacy (RRF preservation, Kt/V)

- Rate of infections and peritonitis

-The absolute change in 24 hour ambulatory BP indexes and aortic systolic BP

-Changes in serum parathormone from baseline

-Changes in the calcium phosphorus product from baseline

- Fracture incidence

- Incidence of joint/muscle pain

*Statistical analysis*

All eligible patients who provided informed consent will be enrolled and randomized to the treatment or placebo group (on a 1:1 ratio), stratified by sex, age and center. All details will be described in a statistical randomization report that will be kept concealed and will be opened only after the enrollment will be completed.

One of the primary endpoints is progression of arterial stiffness, defined as the absolute change in PWV value at the end of the 18 months versus the baseline. We hypothesize that the progression – increase of PWV will be lower in the treatment group (20% lower). Therefore, this endpoint will be the PWV increase for every patient (expressed as absolute value and as percentage change over time). To investigate the possible effect of MK-7 supplementation on the other primary endpoint (CV events) Kaplan-Meier curves and Cox regression analysis will be performed.

*Sample size calculation*

We performed two separate power calculations for our two separate, primary endpoints. The only published study so far assessing dp-ucMGP and clinical hard end-points in PD patients, showed that after a median follow-up of 31 months, a significantly higher incidence of fatal/non-fatal CV events occurred in the patients with high dp-ucMGP levels (64.6% versus 39%). Until to-date no RCTs have been conducted in PD patients, therefore our calculation will be based on already published data in similar populations. We consider that a 28% difference in the incidence of CV events among arms is a significant treatment effect and we expect that the incidence of CV events would be 56% in the placebo and 28% in the treatment group, during the 18 month follow-up. Therefore, with a two-sided significance level of 5%, t-test and 80% power, 96 participants will be required. Accounting for a drop-out of 20%, we will enroll 120 patients (60 in each arm).

For the primary outcome of arterial stiffness progression we performed a separate power analysis. Based on previous studies, we expect that the increase in PWV value (absolute difference from baseline to the end of the study) will be 3 m/s after 1.5 year at the placebo group with an assumed standard deviation of 0.90 m/s (3, 30, 31) and we consider that an absolute difference of 20% in PWV increase among groups, is a significant treatment effect (32, 33). Accounting for an estimating drop-out rate of 20%, with a two-sided significance level of 5%, t-test and 80% power, 120 patients will be required (60 in each group) to detect a significant treatment difference.

*Monitoring*

During the study period, 6 follow-up visits will take place, at months 3, 6, 9, 12, 15 and 18 respectively. In all these visits, patients will be interviewed regarding the compliance and potential side effects (and pills will be counted), and clinical examination and routine blood sampling will be performed. To evaluate the patients’ compliance, the drug boxes will be thoroughly checked and remaining pills will be counted and reported. As before enrollment, all patients will undergo standard, regular follow-up visits every month in their PD unit. At the end of the follow-up period, all plasma and serum parameters that were measured at baseline, along with PWV will be re-assessed. The occurrence of the trial’s endpoints will be documented by death certificates, medical files and records and integrated interview at the last visit or telephone interviews. During the study period, all potential adverse events will be closely monitored and recorded. These data will be evaluated thoroughly. The criteria for early termination of the trial will be withdraw of the written consent, death, kidney transplantation, severe allergic response to MK-7 and medical necessity for treatment initiation with vitamin K antagonists.

**Acknowledgments**

The VIKIPEDIA researchers would like to thank Nattopharma ASA, Hovik, Norway for supporting our study and providing the MK-7 supplements.

**Table 1.** Inclusion and exclusion criteria of the VIKIPEDIA study.

| **Inclusion and exclusion criteria** | |
| --- | --- |
| Inclusion criteria | Exclusion criteria |
| Age ≥ 18 years | Use of vitamin K antagonist or vitamin K supplements during the past 3 months |
| At least 3 months on PD | Liver disease |
| Life expectancy of ≥ 18 months | Drug or alcohol abuse |
|  | Ongoing malignancy or severe inflammatory disease diagnosis |
|  | Diagnosis of severe gut-disease (inflammatory or short bowel disease) or gastrointestinal malabsorption |
|  | Mental disorder rendering the patient unable to conform with the instructions and fully understand the nature, aim and possible side-effects of the supplementation |
|  | Treatment with phosphate binders (sevelamer) |
|  | Pregnancy or breast-feeding |

**Figure 1.** Design and flow-chart of the VIKIPEDIA trial.


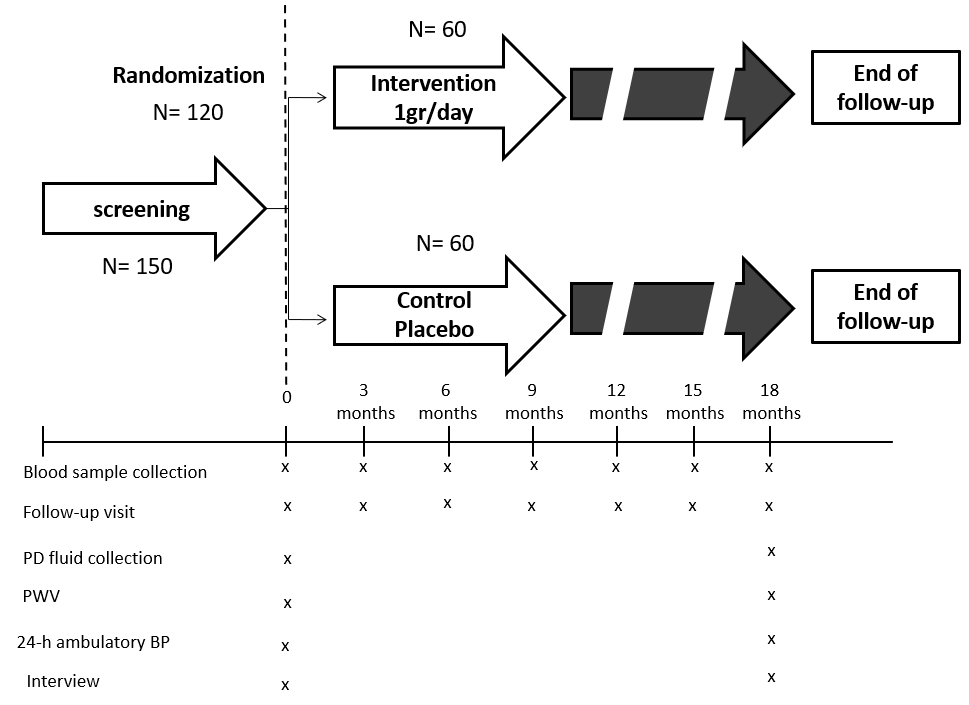


**References**

1. Blacher J, Safar ME, Pannier B, Guerin AP, Marchais SJ, London GM. Prognostic significance of arterial stiffness measurements in end-stage renal disease patients. Current opinion in nephrology and hypertension. 2002;11(6):629-34.

2. Alexandrou M-E, Loutradis C, Balafa O, Theodorakopoulou M, Tzanis G, Bakaloudi D, et al. A comparative study of ambulatory central hemodynamics and arterial stiffness parameters in peritoneal dialysis and hemodialysis patients. Journal of Hypertension. 2020;38(12):2393-403.

3. Levy-Schousboe K, Frimodt-Møller M, Hansen D, Peters CD, Kjærgaard KD, Jensen JD, et al. Vitamin K supplementation and arterial calcification in dialysis: results of the double-blind, randomised, placebo-controlled RenaKvit trial. Clinical Kidney Journal. 2021.

4. Rodriguez RA, Spence M, Hae R, Agharazii M, Burns KD. Pharmacologic therapies for aortic stiffness in end-stage renal disease: a systematic review and meta-analysis. Canadian journal of kidney health and disease. 2020;7:2054358120906974.

5. Luo G, Ducy P, McKee MD, Pinero GJ, Loyer E, Behringer RR, et al. Spontaneous calcification of arteries and cartilage in mice lacking matrix GLA protein. Nature. 1997;386(6620):78-81.

6. Roumeliotis S, Roumeliotis A, Dounousi E, Eleftheriadis T, Liakopoulos V. Biomarkers of vascular calcification in serum. Advances in clinical chemistry. 2020;98:91-147.

7. Roumeliotis S, Dounousi E, Salmas M, Eleftheriadis T, Liakopoulos V. Vascular Calcification in Chronic Kidney Disease: The Role of Vitamin K- Dependent Matrix Gla Protein. Front Med (Lausanne). 2020;7:154.

8. Schurgers LJ, Teunissen KJ, Hamulyak K, Knapen MH, Vik H, Vermeer C. Vitamin K-containing dietary supplements: comparison of synthetic vitamin K1 and natto-derived menaquinone-7. Blood. 2007;109(8):3279-83.

9. Roumeliotis S, Dounousi E, Eleftheriadis T, Liakopoulos V. Association of the Inactive Circulating Matrix Gla Protein with Vitamin K Intake, Calcification, Mortality, and Cardiovascular Disease: A Review. Int J Mol Sci. 2019;20(3).

10. Fain ME, Kapuku GK, Paulson WD, Williams CF, Raed A, Dong Y, et al. Inactive Matrix Gla Protein, Arterial Stiffness, and Endothelial Function in African American Hemodialysis Patients. Am J Hypertens. 2018.

11. Puzantian H, Akers SR, Oldland G, Javaid K, Miller R, Ge Y, et al. Circulating Dephospho-Uncarboxylated Matrix Gla-Protein Is Associated With Kidney Dysfunction and Arterial Stiffness. Am J Hypertens. 2018;31(9):988-94.

12. O'Donnell CJ, Shea MK, Price PA, Gagnon DR, Wilson PW, Larson MG, et al. Matrix Gla protein is associated with risk factors for atherosclerosis but not with coronary artery calcification. Arterioscler Thromb Vasc Biol. 2006;26(12):2769-74.

13. Schurgers LJ, Barreto DV, Barreto FC, Liabeuf S, Renard C, Magdeleyns EJ, et al. The circulating inactive form of matrix gla protein is a surrogate marker for vascular calcification in chronic kidney disease: a preliminary report. Clin J Am Soc Nephrol. 2010;5(4):568-75.

14. Roumeliotis S, Roumeliotis A, Stamou A, Leivaditis K, Kantartzi K, Panagoutsos S, et al. The Association of dp-ucMGP with Cardiovascular Morbidity and Decreased Renal Function in Diabetic Chronic Kidney Disease. Int J Mol Sci. 2020;21(17).

15. Schlieper G, Westenfeld R, Kruger T, Cranenburg EC, Magdeleyns EJ, Brandenburg VM, et al. Circulating nonphosphorylated carboxylated matrix gla protein predicts survival in ESRD. J Am Soc Nephrol. 2011;22(2):387-95.

16. Xu Q, Guo H, Cao S, Zhou Q, Chen J, Su M, et al. Associations of vitamin K status with mortality and cardiovascular events in peritoneal dialysis patients. International urology and nephrology. 2019;51(3):527-34.

17. Roumeliotis S, Roumeliotis A, Dounousi E, Eleftheriadis T, Liakopoulos V. Vitamin K for the treatment of cardiovascular disease in End-Stage Renal Disease patients: is there hope? Curr Vasc Pharmacol. 2020.

18. Roumeliotis S, Roumeliotis A, Eleftheriadis T, Liakopoulos V. Letter to the Editor regarding “Six months vitamin K treatment does not affect systemic arterial calcification or bone mineral density in diabetes mellitus 2”. European Journal of Nutrition. 2021:1-2.

19. Caluwe R, Vandecasteele S, Van Vlem B, Vermeer C, De Vriese AS. Vitamin K2 supplementation in haemodialysis patients: a randomized dose-finding study. Nephrol Dial Transplant.29(7):1385-90.

20. Oikonomaki T, Papasotiriou M, Ntrinias T, Kalogeropoulou C, Zabakis P, Kalavrizioti D, et al. The effect of vitamin K2 supplementation on vascular calcification in haemodialysis patients: a 1-year follow-up randomized trial. International urology and nephrology. 2019;51(11):2037-44.

21. Westenfeld R, Krueger T, Schlieper G, Cranenburg EC, Magdeleyns EJ, Heidenreich S, et al. Effect of vitamin K2 supplementation on functional vitamin K deficiency in hemodialysis patients: a randomized trial. Am J Kidney Dis. 2012;59(2):186-95.

22. Aoun M, Makki M, Azar H, Matta H, Chelala DN. High Dephosphorylated-Uncarboxylated MGP in Hemodialysis patients: risk factors and response to vitamin K2, A pre-post intervention clinical trial. BMC Nephrol. 2017;18(1):191.

23. Chan A-W, Tetzlaff JM, Gøtzsche PC, Altman DG, Mann H, Berlin JA, et al. SPIRIT 2013 explanation and elaboration: guidance for protocols of clinical trials. Bmj. 2013;346.

24. Sabrina-Wong-Peixin Haroon B-C, Tai L-HL, Lynette Teo AD, Leon Schurgers B-WT, Priyanka Khatri C-CO, Sanmay Low X-EY, et al. Treatment to reduce vascular calcification in hemodialysis patients using vitamin K (Trevasc-HDK): a study protocol for a randomized controlled trial. Medicine. 2020;99(36).

25. DeLoach SS, Townsend RR. Vascular stiffness: its measurement and significance for epidemiologic and outcome studies. Clinical Journal of the American Society of Nephrology. 2008;3(1):184-92.

26. Laurent S, Cockcroft J, Van Bortel L, Boutouyrie P, Giannattasio C, Hayoz D, et al. Expert consensus document on arterial stiffness: methodological issues and clinical applications. European heart journal. 2006;27(21):2588-605.

27. Vaios V, Georgianos PI, Pikilidou MI, Eleftheriadis T, Zarogiannis S, Papagianni A, et al., editors. Accuracy of a Newly-Introduced Oscillometric Device for the Estimation of Arterial Stiffness Indices in Patients on Peritoneal Dialysis: A Preliminary Validation Study. Advances in peritoneal dialysis Conference on Peritoneal Dialysis; 2018.

28. Townsend RR, Wilkinson IB, Schiffrin EL, Avolio AP, Chirinos JA, Cockcroft JR, et al. Recommendations for improving and standardizing vascular research on arterial stiffness: a scientific statement from the American Heart Association. Hypertension. 2015;66(3):698-722.

29. Vaios V, Georgianos PI, Vareta G, Dounousi E, Dimitriadis C, Eleftheriadis T, et al. Clinic and home blood pressure monitoring for the detection of ambulatory hypertension among patients on peritoneal dialysis. Hypertension. 2019;74(4):998-1004.

30. Frimodt-Møller M, Nielsen AH, Kamper A-L, Strandgaard S. Reproducibility of pulse-wave analysis and pulse-wave velocity determination in chronic kidney disease. Nephrology Dialysis Transplantation. 2008;23(2):594-600.

31. Blacher J, Safar ME, Guerin AP, Pannier B, Marchais SJ, London GM. Aortic pulse wave velocity index and mortality in end-stage renal disease. Kidney international. 2003;63(5):1852-60.

32. Krueger T, Schlieper G, Schurgers L, Cornelis T, Cozzolino M, Jacobi J, et al. Vitamin K1 to slow vascular calcification in haemodialysis patients (VitaVasK trial): a rationale and study protocol. Nephrol Dial Transplant. 2014;29(9):1633-8.

33. Peeters F, van Mourik MJW, Meex SJR, Bucerius J, Schalla SM, Gerretsen SC, et al. Bicuspid Aortic Valve Stenosis and the Effect of Vitamin K2 on Calcification Using (18)F-Sodium Fluoride Positron Emission Tomography/Magnetic Resonance: The BASIK2 Rationale and Trial Design. Nutrients. 2018;10(4).

**SUPLLEMENT 1 - Consent form**

**Informed Consent form for the Vitamin K In PEritonial DIAlysis (VIKIPEDIA) study**

This informed consent is for peritoneal dialysis patients attended in the peritoneal dialysis units of three, university, tertiary hospitals (1^st^ Division of Internal Medicine, Department of Nephrology and Hypertension, AHEPA hospital, Thessaloniki, Greece, the Nephrology Clinic, University hospital of Ioannina, Greece and the Nephrology Clinic, University Hospital of Alexandroupolis, Greece) and who we are inviting to participate in research on our research project, entitled “Vitamin K In PEritonial DIAlysis (VIKIPEDIA) study”

**Principal Investigator:** Dr. Stefanos Roumeliotis

**Organization:** Division of Nephrology and Hypertension, 1st Department of Medicine, AHEPA Hospital, Aristotle University of Thessaloniki, Greece

**Sponsor:** Nattopharma, ASA, Hovik, Norway

**Proposal and version:** Vitamin K In PEritonial DIAlysis (VIKIPEDIA) study, version 1.0

**This Informed Consent Form has two parts:**

- **Information Sheet (to share information about the research with you)**
- **Certificate of Consent (for signatures if you agree to take part)**

**You will be given a copy of the full Informed Consent Form**

**PART I: Information Sheet**

**Introduction**

I am Dr. Stefanos Roumeliotis, M.D, PhD, FERA-EDTA, working as an Academic Fellow in the Peritoneal Dialysis Unit of Division of Nephrology and Hypertension, 1st Department of Medicine, AHEPA Hospital, Aristotle University of Thessaloniki, Greece. We are doing a research in vascular calcification and stiffness in peritoneal dialysis patients, which is very common in these patients and predispose to cardiovascular disease. We will provide you with all the information needed and we invite you to participate in this study. You do not have to decide today whether or not you will participate in the research. Before you decide, you can talk to anyone you feel comfortable with about the research.

There may be some words that you do not understand. Please ask me to stop as we go through the information and I will take time to explain. If you have questions later, you can ask me, the study doctors or the staff.

**Purpose of the research**

The presence of calcium in the arteries (termed arterial calcification) is very common in peritoneal dialysis patients and is dangerous because predisposes to acute myocardial infractions and strokes. Until today there are no specific drugs that could delay this process. Vitamin K is an over the counter supplement that is used by millions of people around the world. Vitamin K2 has no side effects and has never caused toxicity, so it is safe for peritoneal dialysis patients. It has been found that vitamin K2 might improve arterial calcification by removing the extra calcium from the arterial wall. There are several studies showing that hemodialysis patients receiving vitamin K2 have no side effects, whereas they might live longer and not present myocardial infarction or stroke episodes. The reason we are doing this research is to found out whether this new supplement, vitamin K2 improves the arterial calcification in peritoneal dialysis patients.

**Type of Research Intervention**

This study will involve daily supplementation with one pill for 1.5 years. At the beginning of the study we will perform an examination to evaluate the stiffness of your arteries and your blood pressure. Every month in your regular visit in the peritoneal dialysis unit we will discuss any concerns you might have.

**Participant selection**

We are inviting all peritoneal dialysis patients attended in our unit to participate in the research on the new supplement for arterial calcification.

**Voluntary Participation**

Your participation in this study is completely voluntary. It is entirely your choice whether you want to participate or not and in the case that you chose not to, all services and medical treatment you are currently receiving in the peritoneal dialysis unit will continue and nothing will change. If you choose to participate, you have the right to change your mind later on and then stop participating**.**

**Information on the Trial Supplement Vitamin K2**

The supplement we are testing is vitamin K2, which already exists in your body. Millions of people are receiving everyday vitamin K2 as a supplement, but now we want to test if this vitamin might improve arterial stiffness in peritoneal dialysis patients. The vitamin K2 has no severe side effects except in 5%, very mild symptoms like nausea. There are no other side effects or risks.

**Procedures and Protocol**

To test if vitamin K2 improves arterial stiffness, we need to compare it with a placebo pill. To do this, we will divide all participants in this study in 2 groups. The selection in groups is made completely by chance, as if by tossing a coin. In one group patients will take vitamin K2 and in the other placebo once daily, after eating. Placebo is an inactive pill containing water and sugar that looks exactly like the real supplement. During the study, every month you will continue to have routine visits in the unit and the medical team will be looking after you very carefully. If in this period you may have any concerns, feel free to talk about it with me or one of the other physicians.

We will take your blood and peritoneal effluent at the beginning and at the end of the study (after 1.5 year) and will be tested to assess your vitamin K status. We will also ask you to wear a device that measures your blood pressure for 24 hours and we will ask you to lie in a bed where we will put an ultrasound in your neck to measure your arterial stiffness at the start and end of the study. This study will last 1.5 years. No additional visits are needed in our unit, except your regular, routine monthly visits.

**Side Effects and Risks**

As already mentioned, vitamin K2 is a natural supplement and not a drug. It can only cause you nausea very rarely and no risks or other side-effects have been ever reported in end-stage kidney disease patients.

**Confidentiality**

The information and data we will collect from participants in this study will be kept confidential. No-one, except the researchers will have access on your data. Any information about you will be coded with a number instead of your name and only the researchers will know what your number is.

**Sharing the Results**

The results of this study will be shared with you, before we will publish them in scientific journals or congresses.

**Right to Refuse or Withdraw**

You do not have to participate in the study if you wish to do so. Also, if you agree to participate, you may change your mind and stop participating at any time you choose. It is entirely your choice and we will respect it.

**Who to Contact**

If you have any questions you may ask them now or later, even after the study has started. At any time, you may contact the following physicians:

Stefanos Roumeliotis, [st_roumeliotis@hotmail.com](mailto:st_roumeliotis@hotmail.com), 2313303855

Vassilios Liakopoulos [liakopul@otenet.gr](mailto:liakopul@otenet.gr), 2313303855

**This proposal has been reviewed and approved by the Ethics Committee/Scientific Council of the Medical School of Aristotle University of Thessaloniki (235/14.05.2021), which is a committee whose task it is to make sure that research participants are protected from harm. If you wish to find about more about the IRB, contact [St. Kyriakidi street 1, 54636, Thessaloniki, 2313303110, ahepahos@n3syzefxis.gov.gr].**

You can ask me any more questions about any part of the research study, if you wish to. Do you have any questions?

**PART II: Certificate of Consent**

I have read the foregoing information, or it has been read to me. I have had the opportunity to ask questions about it and any questions that I have asked have been answered to my satisfaction. I consent voluntarily to participate as a participant in this research.

Print Name of Participant__________________

Signature of Participant ___________________

Date ___________________________

Day/month/year

**Statement by the researcher/person taking consent**

**I have accurately read out the information sheet to the potential participant, and to the best of my ability made sure that the participant understands the procedures and protocol.** **I confirm that the participant was given an opportunity to ask questions about the study, and all the questions asked by the participant have been answered correctly and to the best of my ability. I confirm that the individual has not been coerced into giving consent, and the consent has been given freely and voluntarily.**

**A copy of this ICF has been provided to the participant.**

**Print Name of Researcher****/person taking the consent________________________**

**Signature of Researcher /person taking the consent__________________________**

**Date ___________________________**

**Day/month/year**
