# Supplementary material for: VItamin K In PEritonial DIAlysis (VIKIPEDIA): Rationale and study protocol for a randomized controlled trial
Source: PLoS One. 2022 Aug 17;17(8):e0273102. doi: 10.1371/journal.pone.0273102 (PMC9384975; doi:10.1371/journal.pone.0273102)
Supplement: S3 File — (DOCX) [file pone.0273102.s003.docx]

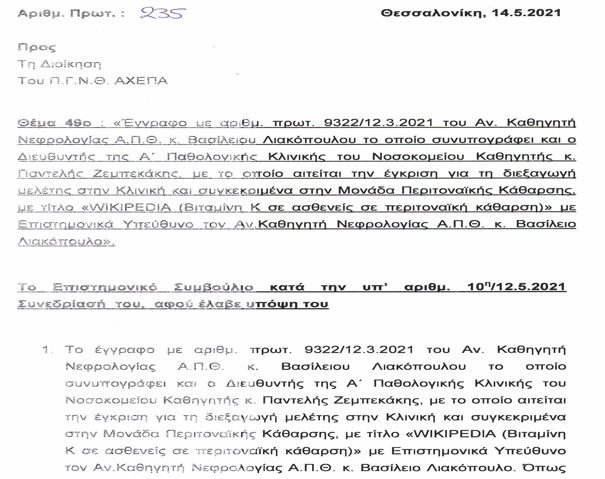


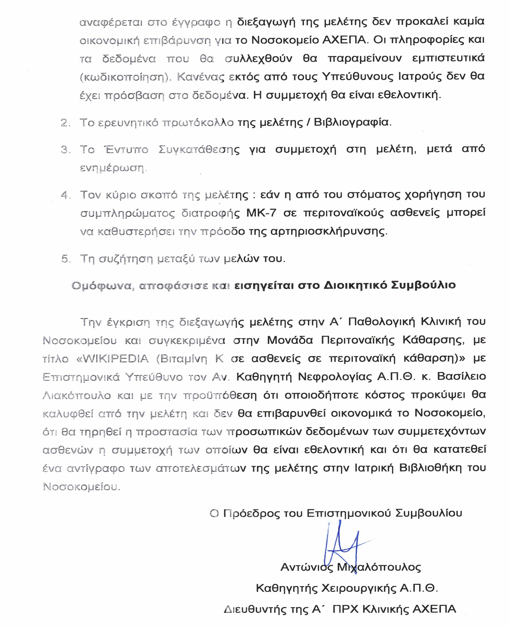


**Περίληψη**

Η αγγειακή επασβέστωση (ΑΕ) είναι μια ενεργή διαδικασία που προκύπτει ως αποτέλεσμα από τη διαταραχή της ισορροπίας μεταξύ αναστολέων και επαγωγέων της επασβέστωσης, προς την πλευρά των επαγωγέων. Η πρωτεΐνη Matrix Gla Protein (MGP), είναι ένας ισχυρός αναστολέας της αγγειακής επασβέστωσης που χρειάζεται βιταμίνη Κ για να γίνει βιολογικά ενεργή. Σε συνθήκες ένδειας βιταμίνης Κ, αυξάνονται τα επίπεδα πλάσματος της ανενεργούς μορφής της MGP, της μη-φωσφορυλιωμένης, μη-καρβοξυλιωμένης MGP(dp-ucMGP) και σχετίζονται άμεσα με την ΑΕ και την καρδιαγγειακή (ΚΑ) νόσο. Οι ασθενείς με Χρόνια Νεφρική Ανεπάρκεια Τελικού Σταδίου (ΧΝΑΤΣ) εμφανίζουν αυξημένα επίπεδα dpucMGP και επιταχυνόμενη ΑΕ. Η μελέτη VIKIPEDIA (VItamin K In PEritoneal DIAlysis) είναι μια προοπτική, τυχαιοποιημένη, ελεγχόμενη κλινική δοκιμή που θα διερευνήσει την επίδραση της χορήγησης βιταμίνης Κ2 στην αρτηριακή σκληρία και τα ΚΑ επεισόδια σε ασθενείς με ΧΝΑΤΣ που υποβάλλονται σε Περιτοναϊκή Κάθαρση (ΠΚ). 120 ΠΚ ασθενείς θα συμπεριληφθούν στη μελέτη. Κατά την ένταξη τους, θα μετρηθεί η αρτηριακή σκληρία (pulse-wave velocity-PWV) και οι ασθενείς θα τυχαιοποιηθούν με αναλογία 1:1 σε δύο ομάδες: την ομάδα ελέγχου που θα λάβει εικονικό φάρμακο και την ομάδα που θα λάβει βιταμίνη Κ (1000 μg μενακουινόνη-7, MK-7/ημέρα) για 18 μήνες. Το κύριο καταληκτικό σημείο της μελέτης είναι η αλλαγή του PWV ανάμεσα στις δυο ομάδες και η εμφάνιση ΚΑ επεισοδίων. Δευτερεύοντα καταληκτικά σημεία είναι η θνητότητα, οι αλλαγές στην επάρκεια κάθαρσης, οι αλλαγές στην 24-ωρη περιπατητική αρτηριακή πίεση και οι αλλαγές στο μεταβολισμό του ασβεστίου/φωσφόρου/παραθορμόνης.

Η μελέτη VIKIPEDIA είναι μια νέα δοκιμή που θα διερευνήσει την επίδραση της καθημερινής χορήγησης βιταμίνης Κ2 στην ΑΕ, την ΚΑ νόσο και το μεταβολισμό του ασβεστίου/φωσφόρου/παραθορμόνης σε ασθενείς υπό ΠΚ. Το πρωτόκολλο της μελέτης είναι καταχωρημένο στο ClinicalTrials.gov με αύξοντα αριθμό NCT04900610.

1. **Εισαγωγή**

Η αυξημένη καρδιαγγειακή νοσηρότητα και θνητότητα των ασθενών με Χρόνια Νεφρική Νόσο (ΧΝΝ) μπορεί να εξηγηθεί εν μέρει από το γεγονός ότι η ΧΝΝ συνιστά μια κατάσταση επιταχυνόμενης αγγειακής επασβέστωσης (ΑΕ) τόσο του μέσου όσο και του έσω τοιχώματος ων αρτηριών. Η ΑΕ του μέσου χιτώνα του αγγειακού τοιχώματος οδηγεί σε σκλήρυνση των αρτηριών, η οποία επιδεινώνεται με την πρόοδο της ΧΝΝ σε Χρόνια Νεφρική Ανεπάρκεια Τελικού Σταδίου (ΧΝΑΤΣ). Ένα δείκτη αρτηριοσκλήρυνσης αποτελεί η ταχύτητα διάδοσης του σφυγμικού κύματος (pulse wave velocity -PWV), που αυξάνεται στην ουραιμία και σχετίζεται με την καρδιαγγειακή νόσο (1). Οι ασθενείς με ΧΝΑΤΣ που υποβάλλονται σε περιτοναϊκή κάθαρση (ΠΚ) σε σχέση με τους ασθενείς που υποβάλλονται σε τεχνητό νεφρό (ΤΝ) εμφανίζουν υψηλότερες τιμές PWV, υποδεικνύοντας έναν υψηλότερο καρδιαγγειακό κίνδυνο στους ασθενείς αυτούς (2). Σε τυχαιοποιημένες κλινικές δοκιμές (ΤΚΔ) σε ασθενείς με ΧΝΑΤΣ, έχουν μελετηθεί διάφορα σκορ ΑΕ. Στην παρούσα μελέτη θα χρησιμοποιήσουμε το PWV ως δείκτη πρώιμης αρτηριακής σκληρίας. Αυτό γιατί τα συγκεκριμένα σκορ πιθανώς αντανακλούν μόνο το τελικό στάδιο της ΑΕ, ενώ το PWV φαίνεται ότι μεταβάλλεται με την πάροδο του χρόνου από τα πρώτα στάδια προοδευτικά προς τη ΧΝΑΤΣ (3). Για αυτό το λόγο, η PWV έχει χρησιμοποιηθεί σε διάφορες μελέτες ως πιθανός δείκτης θεραπευτικής παρέμβασης με σκοπό τη βελτίωση του καρδιαγγειακού κινδύνου σε ασθενείς με ΧΝΑΤΣ (4).

Για μεγάλο χρονικό διάστημα, η ΑΕ θεωρούνταν μια παθητική, εκφυλιστική διαδικασία συγκέντρωσης ασβεστίου στο αρτηριακό τοίχωμα. Η θεώρηση αυτή έχει αλλάξει τις τελευταίες δεκαετίες, καθώς διαπιστώθηκε ότι η ΑΕ συνιστά μια ενεργητική διαδικασία που ρυθμίζεται από αναστολείς και επαγωγείς. Ανάμεσα σε αυτούς, η πρωτεϊνη Matrix Gla Protein (MGP) αποτελεί τον πιο ισχυρό φυσικό αναστολέα της ΑΕ στον οργανισμό. Ο κεντρικός παθοφυσιολογικός ρόλος της MGP επισημάνθηκε πρώτα σε νοκ-αουτ πειραματικά μοντέλα με πλήρη έλλειψη της πρωτεΐνης (MGP -/-), τα οποία πέθαναν 8 εβδομάδες από τη γέννησή τους λόγω της σημαντικής αορτικής επασβέστωσης, η οποία οδήγησε σε ρήξη των αγγείων (5). Η ενεργοποίηση της MGP πραγματοποιείται αρχικώς μέσω της καρβοξυλίωσής της παρουσία της βιταμίνης Κ2 και κατόπιν μέσω της φωσφορυλίωσής της όπου κι εδώ απαιτείται η Κ2. Μόνο μετά από αυτές τις διαδικασίες μπορεί η MGP να δράσει προστατευτικά εναντίον της ΑΕ (6). Σε περίπτωση ένδειας βιταμίνης Κ, πειραματικές και κλινικές μελέτες έδειξαν ότι οι συγκεντρώσεις της μη-ενεργούς, μη-φωσφορυλιωμένης, μη-καρβοξυλιωμένης MGP (dp-ucMGP) στην κυκλοφορία είναι υψηλές (7). Δεδομένα από in vivo και in vitro μελέτες υποδεικνύουν ότι συγκριτικά με τη βιταμίνη K1, η μενακινόνη-7 (menaquinone-7, MK-7), μια ισομορφή της K2 με μακρά άλυσο, έχει αρκετά μεγαλύτερο χρόνο ημίσειας ζωής, υψηλότερη βιοδιαθεσιμότητα και βιοενεργότητα (8) με αποτέλεσμα η χορήγησή της να προτιμάται σε σχέση με την Κ1 στις ΤΚΔ.

Η ΑΕ και η ανεπάρκεια βιταμίνης Κ πιστεύεται ότι είναι αλληλένδετες οντότητες που ξεκινούν ακόμα από τα πρώιμα (1^ο^ και 2^ο^) στάδια της ΧΝΝ. Με την πάροδο του χρόνου οι διαδικασίες αυτές επιτείνονται μαζί με την πρόοδο της νόσου στα στάδια 3 και 4 και επιδεινώνονται έτι περαιτέρω στο τελικό στάδιο (5^ο^), αυτό της ΧΝΑΤΣ (9). Σε ασθενείς με ΧΝΝ και υπό ΤΝ, η dpuc-MGP έχει επανειλημμένως συσχετιστεί με ποικίλους δείκτες ΑΕ και αρτηριοσκλήρυνσης, όπως το PWV (10, 11). Άλλα δεδομένα υποδεικνύουν μια στενή συσχέτιση μεταξύ της dp-ucMGP, της θνησιμότητας και της καρδιαγγειακής νοσηρότητας σε ασθενείς με προχωρημένο στάδιο ΧΝΝ (12-14), σε ασθενείς με ΧΝΑΤΣ και υπό ΤΝ (15) καθώς και υπό ΠΚ (16). Βέβαια αξίζει να σημειωθεί πως από τις μελέτες αυτές μόνο μία από τους Xu *et al*., διενεργήθηκε σε ασθενείς υπό ΠΚ.

Όλο και περισσότερα δεδομένα υποδεικνύουν τη στενή συσχέτιση μεταξύ ανεπάρκειας βιταμίνης Κ και ΑΕ στην ουραιμία. Έτσι, διάφορες ΤΚΔ μελετούν τον πιθανό θεραπευτικό ρόλο της χορήγησης MK-7 στην ΑΕ σε ασθενείς με ΧΝΑΤΣ (17) και υπό ΤΝ (VitaVasK, Trevasc-HDK and Aortic Valve DECalcification trials). Στον ιστότοπο «*clinicaltrials.gov»* υπάρχουν 17 αποτελέσματα στην αναζήτηση του όρου «βιταμίνη Κ» (“vitamin K”) και «αιμοκάθαρση» (“hemodialysis”) και αφορούν σε ΤΚΔ που έχουν τελειώσει ή βρίσκονται σε εξέλιξη (έλεγχος στις 23/02/2021). Παρ’ όλα αυτά, όλες οι μελέτες χορήγησης ΜΚ-7 έχουν πραγματοποιηθεί σε ασθενείς με ΧΝΝ πριν την ένταξη σε αιμοκάθαρση και/ή σε ασθενείς υπό ΤΝ, ενώ καμία από αυτές τις μελέτες δεν έχει πραγματοποιηθεί σε ασθενείς υπό ΠΚ. Επιπλέον, η πλειονότητα αυτών των μελετών εξετάζουν μόνο δείκτες ΑΕ και όχι «σκληρά» καταληκτικά κλινικά σημεία, όπως η θνησιμότητα και τα καρδιαγγειακά συμβάματα, ενώ καμία μελέτη μέχρι σήμερα δεν έχει μελετήσει τον πιθανό ρόλο της MK-7 στην 24ωρη αρτηριακή πίεση (ΑΠ). Ένα άλλο ζήτημα στις ΤΚΔ που πραγματοποιούνται σήμερα σε ασθενείς υπό ΤΝ είναι ότι η χορηγούμενη ημερήσια δόση της MK-7 δεν υπερβαίνει τα 500 μg/ημέρα (18). Η ακριβής δοσολογία της MK-7 που απαιτείται για να αποκαταστήσει την έλλειψη της βιταμίνης K και επομένως να ενεργοποιήσει πλήρως την MGP σε ασθενείς με ΧΝΑΤΣ δεν έχει ακόμα διασαφηνιστεί. Παρά ταύτα, σε μια μελέτη σε ασθενείς υπό ΤΝ που προσπάθησε να απαντήσει σε αυτό το ερώτημα, οι Caluwe *et al.* έδειξαν ότι η ημερήσια χορήγηση 463 μg MK-7 μείωσε μόνο κατά 46% τα επίπεδα της dp-ucMGP και επομένως η δοσολογία αυτή χαρακτηρίστηκε ως υπο-θεραπευτική (19). Η χορήγηση MK-7 σε δόσεις κάτω από 463 μg/ημέρα σε ασθενείς υπό ΤΝ δεν έδειξε κάποιο ευεργετικό αποτέλεσμα στην ΑΕ σε μια πρόσφατη ΤΚΔ (20). Με βάση αυτά τα αποτελέσματα, σε μια άλλη ΤΚΔ σε ασθενείς υπό ΤΝ που πραγματοποιείται τώρα η προτεινόμενη δόση της MK-7 είναι πολύ υψηλότερη, ήτοι 2 g, 3 φορές εβδομαδιαίως (αριθμός μελέτης *NCT04539418).* Η μελέτη PEritoneal DIAlysis (VIKIPEDIA) θα εξετάσει αν η χορήγηση per os υψηλής δόσης (1mg/ημέρα) MK-7 μπορεί να αυξήσει την ενεργοποίηση της MGP, να καταστείλει την dp-ucMGP και επομένως να μειώσει την αρτηριοσκλήρυνση και τον κίνδυνο εμφάνισης καρδιαγγειακής νόσου σε ασθενείς υπό ΠΚ.

Είναι γνωστό ότι η χορήγηση βιταμίνης K2 από του στόματος μπορεί να αυξήσει την καρβοξυλίωση και φωσφορυλίωση της MGP και επομένως να μειώσει τα κυκλοφορούντα επίπεδα της dp-ucMGP. Από τα διάφορα είδη βιταμίνης Κ που υπάρχουν, η βιταμίνη K2 (μενακινόνη-7, MK-7) είναι η πρώτη που κυκλοφόρησε, έχει εγκριθεί και ελεγχθεί ως θεραπεία υποκατάστασης σε ανεπάρκεια βιταμίνης Κ (MenaQ7 ®, Nattopharma, ASA, Hovik, Norway), εμφανίζει αποδεδειγμένη αποτελεσματικότητα στη μείωση της dp-ucMGP (21) και έχει χρησιμοποιηθεί σε διάφορες κλινικές μελέτες σε ασθενείς υπό ΤΝ (19, 22). Η βιταμίνη Κ2 είναι ένα φυσικό συμπλήρωμα διατροφής και σε καμία περίπτωση φάρμακο, που μπορεί να αγοραστεί «πάνω από τον πάγκο» σε φαρμακεία ή σούπερ μάρκετ χωρίς ιατρική συνταγή. Λόγω των πιθανών, πολλαπλών ευεργετικών επιδράσεων της βιταμίνης Κ2, εκατομμύρια άνθρωποι ανα τον κόσμο λαμβάνουν αυτό το συμπλήρωμα διατροφής, χωρίς να έχει αναφερθεί ποτέ τοξικότητα ή παρενέργειες, εκτός από ήπια γαστρεντερολογικά ενοχλήματα. Στη μελέτη VIKIPEDIA θα χορηγήσουμε από του στόματος υψηλές ημερήσιες δόσεις MK-7 σε ασθενείς υπό ΠΚ. Παρά το ότι διάφορες κλινικές δοκιμές έχουν χορηγήσει δόσεις MK-7 από 200-500 μg/ημέρα, αυτή είναι η πρώτη ΤΚΔ όπου η χορηγούμενη δόση της σε περιτοναϊκούς ασθενείς θα φτάσει το 1g ημερησίως. Δεν υφίστανται ανησυχίες αναφορικά με την ασφάλειά της, γιατί άλλες ΤΚΔ έχουν ήδη χορηγήσει παρόμοια δόση ΜΚ-7 σε ασθενείς με ΧΝΑΤΣ, χωρίς να αναφερθούν θρομβωτικά συμβάματα ή παρενέργειες (22).

Επομένως, καθώς εμφανίζουν εμφανώς μειωμένα επίπεδα Κ2 συγκριτικά με τους προ-ενταξιακούς ασθενείς με ΧΝΝ, πιστεύουμε ότι η συγκεκριμένη υψηλή δοσολογία που προτείνουμε για τους περιτοναϊκούς ασθενείς είναι απολύτως αιτιολογημένη, καθώς το πιθανό όφελος και οι ευεργετικές της επιδράσεις μάλλον θα υπερτερούν σε σχέση με την εμφάνιση πιθανών παρενεργειών. Τέλος, καμία μελέτη μέχρι στιγμής δεν έχει αξιολογήσει τη φαρμακοκινητική και φαρμακοδυναμική της dp-ucMGP στους περιτοναϊκούς ασθενείς και τη συγκέντρωση των επιπέδων της dp-ucMGP στο περιτοναϊκό διάλυμα. Για τους παραπάνω λόγους, η προτεινόμενη μελέτη VIKIPEDIA είναι καινοτόμος και επίκαιρη.

1. **Στόχοι της μελέτης**

Ο κύριος σκοπός της μελέτης VIKIPEDIA είναι εάν η από του στόματος χορήγηση ΜΚ-7 σε περιτοναϊκούς ασθενείς μπορεί να καθυστερήσει την πρόοδο της αρτηριοσκλήρυνσης. Αναμένουμε ότι η χορήγηση ΜΚ-7 θα ενισχύσει την καρβοξυλίωση και τη φωσφορυλίωση της MGP, θα καταστείλει την κυκλοφορούσα dp-ucMGP, θα καθυστερήσει την πρόοδο της αρτηριοσκλήρυνσης (όπως θα εκτιμηθεί από την αύξηση του PWV) και επομένως θα μειώσει τον κίνδυνο εμφάνισης καρδιαγγειακών συμβαμάτων. Επιπλέον ερωτηματικά που θα απαντήσει αυτή η μελέτη είναι το αν θα μπορέσει η χορηγούμενη δόση ΜΚ-7 να μειώσει τη θνησιμότητα τόσο από τα καρδιαγγειακά όσο και από όλα τα αίτια και το αν θα βελτιώσει τις παραμέτρους της 24ωρης ΑΠ. Ακόμα, η μελέτη θα αξιολογήσει στοιχεία διατομής (cross-sectional data) σχετικά με τον επιπολασμό της αρτηριοσκλήρυνσης και της ανεπάρκειας της βιταμίνης Κ στους περιτοναϊκούς ασθενείς, καθώς και προοπτικά δεδομένα σχετικά με την ανάπτυξη αρτηριοσκλήρυνσης στους περιτοναϊκούς ασθενείς που δε θα λάβουν τη βιταμίνη ΜΚ-7 (ομάδα εικονικού φαρμάκου).

1. **Υλικά και Μέθοδοι**

*Σχεδιασμός τη μελέτης*

Η VIKIPEDIA αποτελεί μια πολυκεντρική, τυχαιοποιημένη κλινική δοκιμή παρέμβασης, ανοιχτής επισήμανσης (open-label) με εικονικό και πραγματικό φάρμακο, σε περιτοναϊκούς ασθενείς. Το πρωτόκολλο της μελέτης βρίσκεται σε αντιστοιχία με τη Διακήρυξη του Ελσίνκι για τα Ανθρώπινα Δικαιώματα (Helsinki Declaration of Human Rights) και τις Αρχές καλής κλινικής πρακτικής (Good Clinical Practice Guidelines) καθώς και με τα καθιερωμένα πρωτόκολλα (Standard Protocol Items): Συστάσεις για μελέτες παρέμβασης (23). Επίσης η μελέτη αυτή εγκρίθηκε από την Επιτροπή Βιοηθικής και Δεοντολογίας του Επιστημονικού Συμβουλίου της Ιατρικής Σχολής του Αριστοτελείου Πανεπιστημίου Θεσσαλονίκης και Το πρωτόκολλο της μελέτης είναι καταχωρημένο στο ClinicalTrials.gov με αύξοντα αριθμό NCT04900610. Στους συμμετέχοντες θα ζητηθεί να υπογράψουν το έγγραφο συγκατάθεσης αφού τους εξηγηθεί η διαδικασία της μελέτης. Τρεις Πανεπιστημιακές Κλινικές σε τεταρτοταγείς Πανεπιστημιακές Νοσοκομειακές δομές της Βορείου Ελλάδος με μεγάλες Μονάδες ΠΚ θα συμμετέχουν στη μελέτη. Ο σχεδιασμός της μελέτης περιγράφεται στο σχήμα 1. Εν συντομία, η ένταξη των ασθενών θα διαρκέσει 1 έτος. Στην έναρξη της μελέτης, όλοι οι ασθενείς που θα πληρούν τα κριτήρια και θα υπογράψουν τη φόρμα συγκατάθεσης και συναίνεσης θα ενταχθούν στη μελέτη και θα υποβληθούν σε εκτίμηση της αρτηριοσκλήρυνσης μέσω μέτρησης του PWV και των επιπέδων της βιταμίνης Κ μέσω μέτρησης των επιπέδων της dp-ucMGP. Πριν την τυχαιοποίηση, θα ληφθεί αίμα (ορός και πλάσμα), καθώς και δείγμα περιτοναϊκού διαλύματος από όλους τους ασθενείς προκειμένου να πραγματοποιηθούν μετρήσεις που θα αφορούν στα εξής: γενική αίματος, ουρία, κρεατινίνη, κάλιο, νάτριο, ασβέστιο, φώσφορο, c-αντιδρώσα πρωτεΐνη, αλκαλική φωσφατάση, αλβουμίνη, παραθορμόνη, 25-ΟΗ-D3, μαγνήσιο, γλυκοζυλιωμένη αιμοσφαιρίνη και θυρεοειδικές ορμόνες. Οι ασθενείς κατόπιν θα τυχαιοποιηθούν σε μία από τις δύο ομάδες για τους επόμενους 18 μήνες, είτε αυτή του εικονικού φαρμάκου είτε αυτή που θα λάβει τη ΜΚ-7. Όπως προαναφέρθηκε, η βιταμίνη Κ2 είναι ένα φυσικό συμπλήρωμα διατροφής και όχι φάρμακο.

Η κατηγοριοποίηση των ασθενών θα γίνει ώστε να διασφαλιστεί ότι οι δύο ομάδες ασθενών δε θα διαφέρουν σημαντικά στα επίπεδα βιταμίνης Κ και στην αρτηριοσκλήρυνση. Παρ’ όλα αυτά, καθώς οι κλινικοί ιατροί που θα εκτιμήσουν το PWV, τις τιμές στην καταγραφή της 24ωρης ΑΠ και τα καταληκτικά σημεία δε θα γνωρίζουν την ομάδα που θα ανήκουν οι ασθενείς (εικονικό ή πραγματικό φάρμακο), δεν τίθεται θέμα μεροληψίας που σχετίζεται με τα δεδομένα (information bias). Μετά την τυχαιοποίηση, οι ασθενείς θα ακολουθήσουν κανονικά το πρόγραμμα παρακολούθησης στη Μονάδα ΠΚ, θα συνεχίσουν να λαμβάνουν τακτικά τη φαρμακευτική τους αγωγή και οι ασθενείς στην ομάδα του πραγματικού φαρμάκου θα λάβουν επιπλέον 1 g βιταμίνης K2 από του στόματος ημερησίως (MenaQ7 ®, Nattopharma, ASA, Hovik, Norway).

*Κριτήρια ένταξης και αποκλεισμού*

Τα κριτήρια ένταξης και αποκλεισμού περιγράφονται στον πίνακα 1.

*Δείκτης ανεπάρκειας βιταμίνης Κ*

Ως δείκτης ανεπάρκειας της βιταμίνης Κ θα χρησιμοποιηθούν τα επίπεδα της dp-ucMGP του πλάσματος κατά την έναρξη και στο τέλος της μελέτης. Πρόσθετες παράμετροι που θα μετρηθούν είναι η συγκέντρωση της βιταμίνης Κ στο πλάσμα, καθώς και οι πρωτεΐνες που παράγονται εν τη απουσία της Κ-ΙΙ (proteins induced by vitamin K absence-II, PIVKA-II). Αμέσως μετά τη λήψη του αίματος των ασθενών, αυτό θα φυγοκεντρείται και το πλάσμα θα αποθηκεύεται αμέσως στους -80°C, μέχρι την αποστολή του μέσα σε ξηρό πάγο για ανάλυση στο Ινστιτούτο VitaK, Maastricht της Ολλανδίας, όπως έχει περιγραφεί σε άλλη μελέτη (24).

*Τονομετρικές μετρήσεις της αρτηριακής σκληρίας και της κεντρικής αορτικής πίεσης με τη συσκευή Sphygmocor*

Μετρήσεις με την τεχνική της τονομετρίας επιπέδωσης (applanation tonometry) από την κερκιδική αρτηρία θα ληφθούν με τη χρήση ενός στυλοειδούς ανιχνευτή υψηλής πιστότητας (SPT-301, Millar Instruments, Houston, TX) συνδεδεμένου με ηλεκτρονικό υπολογιστή καθώς και του αντίστοιχου λογισμικού Sphygmocor (ArtCor, Sydney, Australia), προκειμένου να εκτιμηθούν οι πιέσεις στην αορτή καθώς και οι τιμές της παραμέτρου AIx. Από το σήμα που λαμβάνεται από την κερκιδική αρτηρία το λογισμικό Sphygmocor ανασυνθέτει την κυματομορφή του αορτικού σφυγμικού κύματος με τη χρήση μίας πιστοποιημένης συνάρτησης γενικού μετασχηματισμού, όπως έχει περιγραφεί αλλού (25). Για τη βαθμονόμηση της κυματομορφής του αορτικού σφυγμικού κύματος, οι τιμές αρτηριακής πίεσης στη βραχιόνιο αρτηρία που μετρώνται με συμβατικό υδραργυρικό σφυγμομανόμετρο αμέσως πριν την καταγραφή με το Sphygmocor θα εισάγονται στο λογισμικό. Η ταχύτητα αγωγής του σφυγμικού κύματος (PWV) μεταξύ καρωτίδας – μηριαίας θα υπολογιστεί με διενέργεια μετρήσεων τονομετρίας επιπέδωσης στην καρωτίδα και τη μηριαία αρτηρία με τον ως άνω στυλοειδή ανιχνευτή (26). Οι κυματομορφές του σφυγμικού κύματος θα αντιπαραβάλλονται με ταυτόχρονα λαμβανόμενο ηλεκτροκαρδιογράφημα και ο χρόνος διάδοσης του σφυγμικού κύματος μεταξύ των δύο θέσεων μέτρησης θα υπολογίζεται από το λογισμικό Sphygmocor με βάση τη χρονική υστέρηση μεταξύ των δύο άκρων ποδών στη μετάδοση του σφυγμικού κύματος από την καρωτίδα/μηραία αρτηρία (27). Η απόσταση κατά μήκος της επιφάνειας του σώματος από τη στερνική εντομή έως το καρωτιδικό σημείο καταγραφής (απόσταση Α) και από τη στερνική εντομή έως το σημείο μέτρησης στη μηριαία (απόσταση Β) θα μετρώνται και η απόσταση διάδοσης του σφυγμικού κύματος θα υπολογίζεται αφαιρώντας την απόσταση Β από την απόσταση Α. Η ταχύτητα αγωγής του σφυγμικού κύματος μεταξύ καρωτίδας και μηριαίας αρτηρίας (PWV) θα υπολογίζεται ως η απόσταση διάδοσης του σφυγμικού κύματος σε μέτρα διηρημένη δια του χρόνου διάδοσης του σφυγμικού κύματος σε δευτερόλεπτα. Θα καταγράφουμε την PWV σε 10 διαδοχικούς καρδιακούς παλμούς, προκειμένου να καλυφθεί ένας πλήρης αναπνευστικός κύκλος. Θα εφαρμοστούν τα κριτήρια ελέγχου ποιότητας της συσκευής για βέλτιστη καταγραφή της κυματομορφής του σφυγμικού κύματος και ο μέσος όρος δύο διαφορετικών καταγραφών θα χρησιμοποιηθεί στη στατιστική ανάλυση (25).

*Περιπατητική καταγραφή της αρτηριακής πίεσης και σκληρίας με τη συσκευή Mobil-O-Graph*

Όλοι οι ασθενείς θα υποβληθούν σε 24ωρη, περιπατητική καταγραφή της βραχιόνιας και αορτικής ΑΠ, καθώς και των δεικτών αρτηριακής σκληρίας με χρήση της συσκευής Mobil-O-Graph (IEM, Stolberg, Germany) (28). Η συσκευή Mobil-O-Graph αποτελεί μια εμπορικά διαθέσιμη ταλαντωσιμετρική συσκευή με βραχιόνια περιχειρίδα, εγκεκριμένη από τον FDA και την ΕΕ και πιστοποιημένη σύμφωνα με τα πρωτόκολλα της Ευρωπαϊκής Εταιρείας Υπέρτασης ως προς τις καταγραφές της βραχιόνιας ΑΠ. Η συσκευή θα προγραμματιστεί να λαμβάνει 3 καταγραφές ανά ώρα κατά την περίοδο της ημέρας (07:00 έως τις 22:59) και 2 καταγραφές ανά ώρα την περίοδο της νύχτας (23:00 έως τις 06:59). Η μεθοδολογία που ενσωματώνει η συσκευή αυτή έχει ήδη περιγραφεί λεπτομερώς στο παρελθόν (29). Εν συντομία, μετά την αρχική συμβατική, ταλαντωσιμετρική μέτρηση της βραχιόνιας ΑΠ, ακολουθεί καταγραφή των βραχιόνιων κυματομορφών πίεσης, διατηρώντας την περιχειρίδα φουσκωμένη στο επίπεδο της διαστολικής ΑΠ για περίπου 10 δευτερόλεπτα. Ακολούθως δημιουργείται μια αορτική κυματομορφή πίεσης μέσω γενικευμένων συναρτήσεων μεταφοράς (ARCSolver algorythm). Η αορτική ΣΑΠ προκύπτει στη συνέχεια ύστερα από βαθμονόμηση της αορτικής κυματομορφής με χρήση της μέσης και διαστολικής ΑΠ. Επίσης, η συσκευή Mobil-O-Graph παρέχει υπολογισμό της PWV χρησιμοποιώντας περίπλοκους μαθηματικούς αλγορίθμους, οι οποίοι λαμβάνουν υπόψη την ηλικία, τα επίπεδα της ΑΠ και άλλες παραμέτρους από την κυματομορφή του σφυγμικού κύματος στην αορτή. Η αορτική ΑΠ και η PWV όπως αξιολογούνται με τη συσκευή Mobil-O-Graph έχουν συγκριθεί σε στατικές συνθήκες με την gold-standard τονομετρική μεθοδολογία (Sphygmocor, Atcor, Sydney, Australia) και έχουν φανεί αξιόπιστες σε ασθενείς με ΧΝΝ τελικού σταδίου υπό αιμοκάθαρση ή περιτοναϊκή κάθαρση. Επίσης, οι μετρήσεις της συσκευής Mobil-O-Graph συγκρίθηκαν με επεμβατικές, ενδο-αορτικές καταγραφές σε ασθενείς με φυσιολογική νεφρική λειτουργία στο πλαίσιο διαγνωστικής στεφανιογραφίας δίνοντας ιδιαίτερα ακριβή αποτελέσματα. Η μέθοδος βαθμονόμησης (μέση και διαστολική ΑΠ) που θα χρησιμοποιηθεί έχει φανεί ότι παρέχει τα πιο αξιόπιστα και ακριβή αποτελέσματα ως προς τη μη επεμβατική εκτίμηση της αορτικής πίεσης και της PWV συγκριτικά με τις ενδο-αορτικές μετρήσεις. Επιπλέον, η συσκευή Mobil-O-Graph παρέχει μετρήσεις με υψηλή επαναληψιμότητα σε περιπατητικές συνθήκες τόσο για τη βραχιόνια, όσο και την αορτική ΑΠ.

*Καταληκτικά σημεία της μελέτης*

Δύο θα είναι τα κύρια καταληκτικά σημεία της μελέτης:

-Η πρόοδος της αρτηριοσκλήρυνσης, όπως αυτή θα αξιολογηθεί από την αλλαγή στην απόλυτη τιμή του PWV, 18 μήνες μετά την έναρξη της χορήγησης τη ΜΚ-7, σε σχέση με την απόλυτη τιμή της κατά την έναρξη της μελέτης, δηλαδή πριν τη χορήγησή της.

-Η εμφάνιση καρδιαγγειακού συμβάματος , όπως οξύ έμφραγμα του μυοκαρδίου, οξύ στεφανιαίο σύνδρομο, εμβολή, περιφερική αρτηριακή νόσος και αγγειακό εγκεφαλικό επεισόδιο.

Ως δευτερογενή καταληκτικά σημεία θα θεωρηθούν τα παρακάτω:

-Η θνησιμότητα λόγω καρδιαγγειακού συμβάματος καθώς και από οποιοδήποτε άλλο αίτιο

- Η απόλυτη αλλαγή σε σχέση με την έναρξη στις τιμές της PWV, στους δείκτες ανάκλασης κύματος, στον κεντρικό αυξητικό δείκτη προσαρμοσμένο για τους καρδιακούς παλμούς (heart-rate-adjusted augmentation index), στο SVRI και PP

-Η ποσοστιαία μεταβολή στους ανωτέρω δείκτες

-Η επάρκεια κάθαρσης στην ΠΚ (Διατήρηση Υπολειπόμενης Νεφρικής Λειτουργίας, υπολογισμός Kt/V)

-Η αλλαγή στην απόλυτη τιμή των δεικτών που υπολογίζονται στην 24ωρη καταγραφή της ΑΠ, καθώς και η αλλαγή στις τιμές της συστολικής ΑΠ

- Αλλαγές στην παραθορμόνη του ορού σε σχέση με τα επίπεδά της στην αρχή της μελέτης

-Αλλαγές στο γινόμενο ασβεστίου-φωσφόρου σε σχέση με την έναρξη της μελέτης

- Εμφάνιση κατάγματος

*Στατιστική Ανάλυση*

Όλοι οι ασθενείς που θα παραχωρήσουν έγγραφο συγκατάθεσης κατόπιν ενημέρωσης θα ενταχθούν και θα τυχαιοποιηθούν σε μία από τις δύο ομάδες ασθενών: στην ομάδα χορήγηση βιταμίνης ΜΚ-7 ή στην ομάδα χορήγησης του εικονικού φαρμάκου-placebo (σε αναλογία 1:1), αναλόγως ηλικίας, φύλου και κέντρου. Όλα τα στατιστικά δεδομένα θα καταγραφούν σε ένα αρχείο το οποίο θα ανοιχτεί μετά το πέρας της ένταξης όλων των ασθενών.

Ένα από τα πρώιμα κλινικά σημεία που θα αξιολογηθούν είναι η πρόοδος της αρτηριοσκλήρυνσης, όπως αυτή ορίζεται από την απόλυτη αλλαγή στην τιμή του PWV στο τέλος των 18 μηνών συγκριτικά με την τιμή του στην αρχή της μελέτης. Υποθέτουμε ότι η αύξηση της τιμής του PWV θα είναι χαμηλότερη στην ομάδα που θα λάβει τη ΜΚ-7 (περίπου κατά 20%). Επομένως, αυτό το καταληκτικό σημείο θα είναι η αύξηση της τιμής της PWV για κάθε ασθενή (που εκφράζεται ως απόλυτη τιμή καθώς και ως ποσοστιαία αλλαγή στην πάροδο του χρόνου). Για να ερευνήσουμε την πιθανή αποτελεσματικότητα της χορήγησης MK-7 στο έτερο καταληκτικό σημείο (κίνδυνος από καρδιαγγειακά συμβάματα) θα εφαρμοστούν καμπύλες Kaplan-Meier και ανάλυση Cox regression.

*Υπολογισμός του μεγέθους του δείγματος*

Πραγματοποιήσαμε δύο ξεχωριστούς υπολογισμούς για το μέγεθος του δείγματος αναφορικά με τα δύο πρωτογενή καταληκτικά σημεία. Υπάρχει μόνο μία μέχρι σήμερα δημοσιευμένη μελέτη σε περιτοναϊκούς ασθενείς που έχει μετρήσει τα επίπεδα της dp-ucMGP και τα έχει συσχετίσει με σκληρά καταληκτικά σημεία. Η μελέτη αυτή παρακολούθησε τους ασθενείς για διάμεσο διάστημα 31 μηνών και κατέγραψε σημαντικά υψηλότερη συχνότητα θανατηφόρων και μη καρδιαγγειακών συμβαμάτων (64,6%) σε αυτούς με τα υψηλότερα επίπεδα dp-ucMGP σε σχέση με τους ασθενείς με τα χαμηλότερα επίπεδα (39%). Μέχρι σήμερα καμία ΤΚΔ δεν έχει διενεργηθεί σε ασθενείς υπό ΠΚ, οπότε οι υπολογισμοί μας θα βασιστούν σε δημοσιευμένα δεδομένα σε παρόμοιους πληθυσμούς. Κατά τη διάρκεια των 18 μηνών της μελέτης μας, θεωρούμε πως μια διαφορά στην εμφάνιση καρδιαγγειακών συμβαμάτων μεταξύ των δύο ομάδων της τάξεως του 28% αποτελεί ένα σημαντικό θεραπευτικό αποτέλεσμα. Αναμένουμε πως η εμφάνιση καρδιαγγειακών συμβαμάτων στην ομάδα ασθενών που θα λάβει το εικονικό φάρμακο θα ανέλθει στο 56%, ενώ στην ομάδα που θα λάβει τη ΜΚ-7 μόλις στο28%. Επομένως, με στατιστική σημαντικότητα (two-sided significance) που ανέρχεται στο 5%, t-test και ισχύ στο 80%, οι συμμετέχοντες που απαιτούνται υπολογίζονται στους 96. Αν υποτεθεί πως το ποσοστό των ασθενών που θα εγκαταλείψουν τη μελέτη (drop-out) θα αγγίξει το 20%, χρειάζεται να εντάξουμε 120 ασθενείς (60 σε κάθε σκέλος/ομάδα ασθενών).

Για το πρωτογενές καταληκτικό σημείο της προόδου της αρτηριοσκλήρυνσης, εφαρμόσαμε μια ξεχωριστή ανάλυση υπολογισμού μεγέθους δείγματος. Με βάση προηγούμενες μελέτες, αναμένουμε ότι η αύξηση στην τιμή του PWV (αλλαγή στην απόλυτη τιμή από την έναρξη έως το πέρας της μελέτης) θα υπολογιστεί στα 3 m/s μετά από 18 μήνες παρακολούθησης στην ομάδα του εικονικού φαρμάκου με τυπική απόκλιση (standard deviation) στα 0.90 m/s (3, 30, 31). Επίσης υπολογίζουμε ότι μια απόλυτη διαφορά μεταξύ των δύο ομάδων ασθενών στην αύξηση της τιμής της PWV της τάξεως του 20%, αποτελεί σημαντικό στοιχείο αποτελεσματικότητας της χορήγησης της ΜΚ-7 (32, 33). Υπολογίζοντας πως το ποσοστό των ασθενών που θα εγκαταλείψουν τη μελέτη (drop-out) θα αγγίξει το 20%, με επίπεδο στατιστικής σημαντικότητας (two-sided significance level) το 5%, t-test και ισχύ 80%, οι ασθενείς που απαιτείται να ενταχθούν στη μελέτη ανέρχονται στους 120 (60 σε κάθε ομάδα) προκειμένου να διαπιστωθεί σημαντικό θεραπευτικό όφελος από τη χορήγηση της ΜΚ-7.

*Παρακολούθηση*

Κατά τη διάρκεια της μελέτης, θα πραγματοποιηθούν 6 επισκέψεις, στους 3, 6, 9, 12, 15 και 18 μήνες. Σε όλες αυτές τις επισκέψεις, οι ασθενείς θα υποβάλλονται σε ερωτηματολόγια που θα αφορούν στη συμμόρφωσή τους στη θεραπεία και σε πιθανές παρενέργειες που μπορεί να εμφάνισαν. Επίσης θα υποβάλλονται σε κλινική εξέταση και θα πραγματοποιείται λήψη αίματος. Προκειμένου να διαπιστωθεί η συμμόρφωσή τους θα ελέγχονται τα κουτιά με τη βιταμίνη ή το εικονικό φάρμακο και τα εναπομείναντα χάπια θα μετρώνται και θα καταγράφονται. Όπως και πριν την ένταξη, όλοι οι ασθενείς θα προσέρχονται για τη συνήθη, τακτική επίσκεψη κάθε μήνα στη Μονάδα Περιτοναϊκής Κάθαρσης. Στο τέλος της περιόδου παρακολούθησης, όλες οι παράμετροι πλάσματος και ορού που θα έχουν μετρηθεί στην αρχή της μελέτης, καθώς και η τιμή του PWV θα επανεκτιμηθούν. Η εμφάνιση των καταληκτικών σημείων της μελέτης θα τεκμηριωθεί με πιστοποιητικά θανάτου, ιατρικά αρχεία και φακέλους, καθώς και μέσω τηλεφωνικής συνέντευξης ή κατά την τελευταία επίσκεψη στα πλαίσια της μελέτης. Κατά τη διάρκεια της μελέτης όλες οι πιθανές παρενέργειες που μπορεί να εμφανιστούν θα παρακολουθούνται στενά, θα καταγράφονται και τα δεδομένα αυτά θα αξιολογηθούν διεξοδικά. Τα κριτήρια για τον πρόωρο τερματισμό της μελέτης θα είναι η ανάκληση της γραπτής συγκατάθεσης, ο θάνατος, η μεταμόσχευση νεφρού, η σοβαρή αλλεργική αντίδραση στη ΜΚ-7 και η ανάγκη για έναρξη θεραπείας με ανταγωνιστή της βιταμίνης Κ.

**Ευχαριστίες**

Οι ερευνητές της μελέτης VIKIPEDIA θα ήθελαν να ευχαριστήσουν την εταιρεία Nattopharma ASA, Hovik, Norway για τη στήριξή τους στην παρούσα μελέτη, καθώς και για τη χορήγηση της από του στόματος βιταμίνης MK-7.

**Πίνακας 1.** Κριτήρια ένταξης και αποκλεισμού από τη μελέτη VIKIPEDIA.

| **Κριτήρια ένταξης και αποκλεισμού** | |
| --- | --- |
| Κριτήρια ένταξης | Κριτήρια αποκλεισμού |
| Ηλικία ≥ 18 ετών | Προϋπάρχουσα λήψη βιταμίνης Κ ή ανταγωνιστή της τους τελευταίους 3 μήνες |
| Τουλάχιστον 3 μήνες υπό ΠΚ | Ηπατική Νόσος |
| Προσδόκιμο ζωής ≥ 18 μήνες | Κατάχρηση πρόσληψης φαρμακευτικών ή ναρκωτικών ουσιών |
|  | Ύπαρξη κακοήθειας ή σοβαρής φλεγμονώδους νόσου |
|  | Ύπαρξη σοβαρής νόσου του γαστρεντερικού (φλεγμονώδης νόσος του εντέρου ή νόσος βραχέος εντέρου) ή γνωστή δυσαπορρόφηση από το γαστρεντερικό |
|  | Νοητική διαταραχή που καθιστά τον ασθενή μη ικανό να ακολουθήσει τις οδηγίες πρόσληψης της βιταμίνης και να αντιληφθεί τη φύση, το στόχο και τους πιθανές παρενέργειες της υποκατάστασης |
|  | Θεραπεία της υπερφωσφαταιμίας (σεβελαμέρη) |
|  | Κύηση ή γαλουχία |

**Πίνακας 1.** Διαγραμματική απεικόνιση του πρωτοκόλλου της μελέτης VIKIPEDIA.


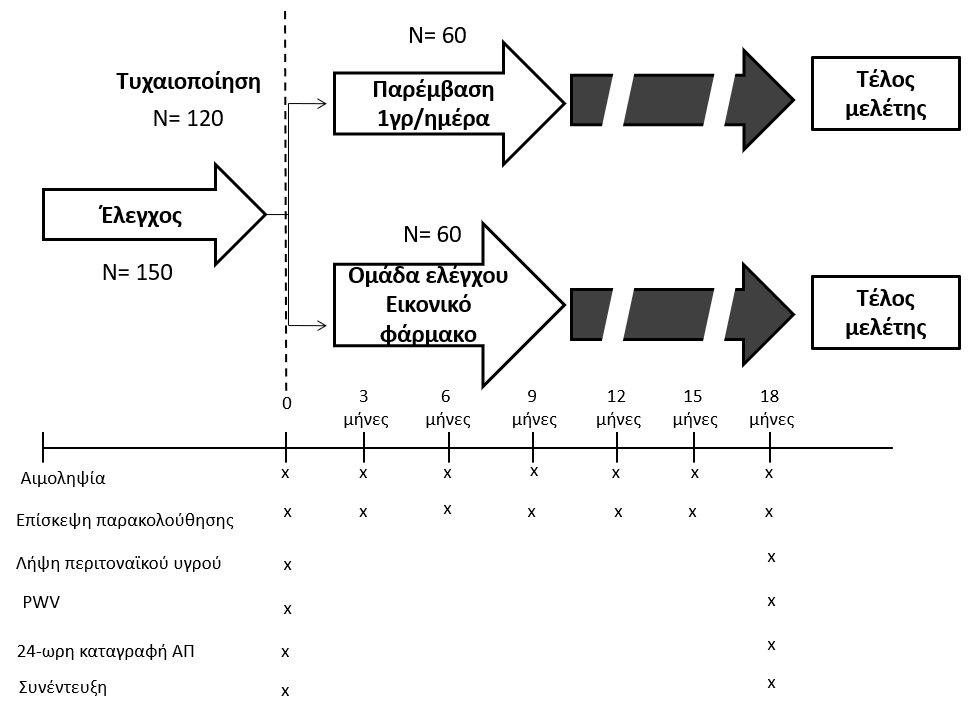


**Βιβλιογραφία**

1. Blacher J, Safar ME, Pannier B, Guerin AP, Marchais SJ, London GM. Prognostic significance of arterial stiffness measurements in end-stage renal disease patients. Current opinion in nephrology and hypertension. 2002;11(6):629-34.

2. Alexandrou M-E, Loutradis C, Balafa O, Theodorakopoulou M, Tzanis G, Bakaloudi D, et al. A comparative study of ambulatory central hemodynamics and arterial stiffness parameters in peritoneal dialysis and hemodialysis patients. Journal of Hypertension. 2020;38(12):2393-403.

3. Levy-Schousboe K, Frimodt-Møller M, Hansen D, Peters CD, Kjærgaard KD, Jensen JD, et al. Vitamin K supplementation and arterial calcification in dialysis: results of the double-blind, randomised, placebo-controlled RenaKvit trial. Clinical Kidney Journal. 2021.

4. Rodriguez RA, Spence M, Hae R, Agharazii M, Burns KD. Pharmacologic therapies for aortic stiffness in end-stage renal disease: a systematic review and meta-analysis. Canadian journal of kidney health and disease. 2020;7:2054358120906974.

5. Luo G, Ducy P, McKee MD, Pinero GJ, Loyer E, Behringer RR, et al. Spontaneous calcification of arteries and cartilage in mice lacking matrix GLA protein. Nature. 1997;386(6620):78-81.

6. Roumeliotis S, Roumeliotis A, Dounousi E, Eleftheriadis T, Liakopoulos V. Biomarkers of vascular calcification in serum. Advances in clinical chemistry. 2020;98:91-147.

7. Roumeliotis S, Dounousi E, Salmas M, Eleftheriadis T, Liakopoulos V. Vascular Calcification in Chronic Kidney Disease: The Role of Vitamin K- Dependent Matrix Gla Protein. Front Med (Lausanne). 2020;7:154.

8. Schurgers LJ, Teunissen KJ, Hamulyak K, Knapen MH, Vik H, Vermeer C. Vitamin K-containing dietary supplements: comparison of synthetic vitamin K1 and natto-derived menaquinone-7. Blood. 2007;109(8):3279-83.

9. Roumeliotis S, Dounousi E, Eleftheriadis T, Liakopoulos V. Association of the Inactive Circulating Matrix Gla Protein with Vitamin K Intake, Calcification, Mortality, and Cardiovascular Disease: A Review. Int J Mol Sci. 2019;20(3).

10. Fain ME, Kapuku GK, Paulson WD, Williams CF, Raed A, Dong Y, et al. Inactive Matrix Gla Protein, Arterial Stiffness, and Endothelial Function in African American Hemodialysis Patients. Am J Hypertens. 2018.

11. Puzantian H, Akers SR, Oldland G, Javaid K, Miller R, Ge Y, et al. Circulating Dephospho-Uncarboxylated Matrix Gla-Protein Is Associated With Kidney Dysfunction and Arterial Stiffness. Am J Hypertens. 2018;31(9):988-94.

12. O'Donnell CJ, Shea MK, Price PA, Gagnon DR, Wilson PW, Larson MG, et al. Matrix Gla protein is associated with risk factors for atherosclerosis but not with coronary artery calcification. Arterioscler Thromb Vasc Biol. 2006;26(12):2769-74.

13. Schurgers LJ, Barreto DV, Barreto FC, Liabeuf S, Renard C, Magdeleyns EJ, et al. The circulating inactive form of matrix gla protein is a surrogate marker for vascular calcification in chronic kidney disease: a preliminary report. Clin J Am Soc Nephrol. 2010;5(4):568-75.

14. Roumeliotis S, Roumeliotis A, Stamou A, Leivaditis K, Kantartzi K, Panagoutsos S, et al. The Association of dp-ucMGP with Cardiovascular Morbidity and Decreased Renal Function in Diabetic Chronic Kidney Disease. Int J Mol Sci. 2020;21(17).

15. Schlieper G, Westenfeld R, Kruger T, Cranenburg EC, Magdeleyns EJ, Brandenburg VM, et al. Circulating nonphosphorylated carboxylated matrix gla protein predicts survival in ESRD. J Am Soc Nephrol. 2011;22(2):387-95.

16. Xu Q, Guo H, Cao S, Zhou Q, Chen J, Su M, et al. Associations of vitamin K status with mortality and cardiovascular events in peritoneal dialysis patients. International urology and nephrology. 2019;51(3):527-34.

17. Roumeliotis S, Roumeliotis A, Dounousi E, Eleftheriadis T, Liakopoulos V. Vitamin K for the treatment of cardiovascular disease in End-Stage Renal Disease patients: is there hope? Curr Vasc Pharmacol. 2020.

18. Roumeliotis S, Roumeliotis A, Eleftheriadis T, Liakopoulos V. Letter to the Editor regarding “Six months vitamin K treatment does not affect systemic arterial calcification or bone mineral density in diabetes mellitus 2”. European Journal of Nutrition. 2021:1-2.

19. Caluwe R, Vandecasteele S, Van Vlem B, Vermeer C, De Vriese AS. Vitamin K2 supplementation in haemodialysis patients: a randomized dose-finding study. Nephrol Dial Transplant.29(7):1385-90.

20. Oikonomaki T, Papasotiriou M, Ntrinias T, Kalogeropoulou C, Zabakis P, Kalavrizioti D, et al. The effect of vitamin K2 supplementation on vascular calcification in haemodialysis patients: a 1-year follow-up randomized trial. International urology and nephrology. 2019;51(11):2037-44.

21. Westenfeld R, Krueger T, Schlieper G, Cranenburg EC, Magdeleyns EJ, Heidenreich S, et al. Effect of vitamin K2 supplementation on functional vitamin K deficiency in hemodialysis patients: a randomized trial. Am J Kidney Dis. 2012;59(2):186-95.

22. Aoun M, Makki M, Azar H, Matta H, Chelala DN. High Dephosphorylated-Uncarboxylated MGP in Hemodialysis patients: risk factors and response to vitamin K2, A pre-post intervention clinical trial. BMC Nephrol. 2017;18(1):191.

23. Chan A-W, Tetzlaff JM, Gøtzsche PC, Altman DG, Mann H, Berlin JA, et al. SPIRIT 2013 explanation and elaboration: guidance for protocols of clinical trials. Bmj. 2013;346.

24. Sabrina-Wong-Peixin Haroon B-C, Tai L-HL, Lynette Teo AD, Leon Schurgers B-WT, Priyanka Khatri C-CO, Sanmay Low X-EY, et al. Treatment to reduce vascular calcification in hemodialysis patients using vitamin K (Trevasc-HDK): a study protocol for a randomized controlled trial. Medicine. 2020;99(36).

25. DeLoach SS, Townsend RR. Vascular stiffness: its measurement and significance for epidemiologic and outcome studies. Clinical Journal of the American Society of Nephrology. 2008;3(1):184-92.

26. Laurent S, Cockcroft J, Van Bortel L, Boutouyrie P, Giannattasio C, Hayoz D, et al. Expert consensus document on arterial stiffness: methodological issues and clinical applications. European heart journal. 2006;27(21):2588-605.

27. Vaios V, Georgianos PI, Pikilidou MI, Eleftheriadis T, Zarogiannis S, Papagianni A, et al., editors. Accuracy of a Newly-Introduced Oscillometric Device for the Estimation of Arterial Stiffness Indices in Patients on Peritoneal Dialysis: A Preliminary Validation Study. Advances in peritoneal dialysis Conference on Peritoneal Dialysis; 2018.

28. Townsend RR, Wilkinson IB, Schiffrin EL, Avolio AP, Chirinos JA, Cockcroft JR, et al. Recommendations for improving and standardizing vascular research on arterial stiffness: a scientific statement from the American Heart Association. Hypertension. 2015;66(3):698-722.

29. Vaios V, Georgianos PI, Vareta G, Dounousi E, Dimitriadis C, Eleftheriadis T, et al. Clinic and home blood pressure monitoring for the detection of ambulatory hypertension among patients on peritoneal dialysis. Hypertension. 2019;74(4):998-1004.

30. Frimodt-Møller M, Nielsen AH, Kamper A-L, Strandgaard S. Reproducibility of pulse-wave analysis and pulse-wave velocity determination in chronic kidney disease. Nephrology Dialysis Transplantation. 2008;23(2):594-600.

31. Blacher J, Safar ME, Guerin AP, Pannier B, Marchais SJ, London GM. Aortic pulse wave velocity index and mortality in end-stage renal disease. Kidney international. 2003;63(5):1852-60.

32. Krueger T, Schlieper G, Schurgers L, Cornelis T, Cozzolino M, Jacobi J, et al. Vitamin K1 to slow vascular calcification in haemodialysis patients (VitaVasK trial): a rationale and study protocol. Nephrol Dial Transplant. 2014;29(9):1633-8.

33. Peeters F, van Mourik MJW, Meex SJR, Bucerius J, Schalla SM, Gerretsen SC, et al. Bicuspid Aortic Valve Stenosis and the Effect of Vitamin K2 on Calcification Using (18)F-Sodium Fluoride Positron Emission Tomography/Magnetic Resonance: The BASIK2 Rationale and Trial Design. Nutrients. 2018;10(4).

**SUPPLEMENT 2- Φόρμα ενημέρωσης και συγκατάθεσης για τη μελέτη VIKIPEDIA (Βιταμίνη Κ σε ασθενείς σε Περιτοναϊκή Κάθαρση)**

Αυτή η φόρμα ενημέρωσης και συγκατάθεσης για συμμετοχή σε μελέτη αφορά ασθενείς υπό Περιτοναϊκή Κάθαρση που παρακολουθούνται στις μονάδες Περιτοναϊκής Κάθαρσης 3 πανεπιστημιακών, τριτοβάθμιων νοσοκομείων (Α' Πανεπιστημιακή Παθολογική Κλινική, Τμήμα Νεφρολογίας και Υπέρτασης, Πανεπιστημιακό Γενικό Νοσοκομείο Θεσσαλονίκης ΑΧΕΠΑ, Θεσσαλονίκη, Πανεπιστημιακή Νεφρολογική Κλινική, Πανεπιστημιακό Γενικό Νοσοκομείο Αλεξανδρούπολης, Αλεξανδρούπολη και Πανεπιστημιακή Νεφρολογική Κλινική, Πανεπιστημιακό Γενικό Νοσοκομείο Ιωαννίνων, Ιωάννινα) τους οποίους καλούμε να συμμετάσχουν στο ερευνητικό πρόγραμμα της μελέτης VIKIPEDIA.

**Κύριος Ερευνητής:** Δρ. Στέφανος Ρουμελιώτης

**Διοργάνωση:** Α' Πανεπιστημιακή Παθολογική Κλινική, Τμήμα Νεφρολογίας και Υπέρτασης, Πανεπιστημιακό Γενικό Νοσοκομείο Θεσσαλονίκης ΑΧΕΠΑ, Θεσσαλονίκη, Ελλάδα

**Σπόνσορας:** Nattopharma, ASA, Hovik, Nορβηγία

**Πρόταση και εκδοχή:** VIKIPEDIA (Βιταμίνη Κ σε ασθενείς σε Περιτοναϊκή Κάθαρση), εκδοχή 1.0

**Αυτή η Φόρμα ενημέρωσης και συγκατάθεσης αποτελείται από δύο μέρη:**

- **Δελτίο πληροφοριών (για να μοιραστούμε πληροφορίες σχετικά με τη μελέτη με εσάς)**
- **Πιστοποιητικό συναίνεσης (για υπογραφές εάν αποφασίσετε να πάρετε μέρος στη μελέτη)**

**Θα σας δοθεί αντίγραφο της Φόρμας ενημέρωσης και συγκατάθεσης**

**ΜΕΡΟΣ I: Δελτίο πληροφοριών**

**Εισαγωγή**

Ονομάζομαι Δρ. Στέφανος Ρουμελιώτης, είμαι ειδικός νεφρολόγος και εργάζομαι ως Ακαδημαϊκός Υπότροφος στην Α' Πανεπιστημιακή Παθολογική Κλινική, Τμήμα Νεφρολογίας και Υπέρτασης, Πανεπιστημιακό Γενικό Νοσοκομείο Θεσσαλονίκης ΑΧΕΠΑ, Θεσσαλονίκη. Διεξάγουμε μια έρευνα για την αγγειακή επασβέστωση σε ασθενείς υπό Περιτοναϊκή Κάθαρση. Σε αυτούς τους ασθενείς η επασβέστωση των αγγείων είναι πολύ συχνή, σε σοβαρό βαθμό και αυξάνει το κίνδυνο για κάποιο καρδιαγγειακό επεισόδιο, όπως οξύ έμφραγμα του μυοκαρδίου ή αγγειακό εγκεφαλικό επεισόδιο. Θα σας παρέχουμε όλες τις απαραίτητες πληροφορίες και σας προσκαλούμε να συμμετάσχετε στη μελέτη αυτή. Δε χρειάζεται να αποφασίσετε σήμερα αν θα συμμετάσχετε στη μελέτη ή όχι. Πριν αποφασίσετε, μπορείτε να μιλήσετε σε οποιονδήποτε αισθάνεστε άνετα σχετικά με τη μελέτη. Μπορεί να υπάρχουν κάποιες λέξεις τις οποίες δεν καταλαβαίνετε. Παρακαλώ, όποτε θέλετε, σταματήστε με και ρωτήστε με όσο εξηγώ τις πληροφορίες αυτές. Ακόμα και αν έχετε απορίες ή ερωτήσεις μετά, μπορείτε να ρωτήσετε εμένα ή οποιονδήποτε νεφρολόγο της Μονάδας Περιτοναϊκής Κάθαρσης σχετικά με τη μελέτη αυτή.

**Σκοπός της μελέτης**

Η παρουσία ασβεστίου στο τοίχωμα των αρτηριών (που ονομάζεται αγγειακή επασβέστωση) είναι πολύ συχνή στους ασθενείς υπό Περιτοναϊκή Κάθαρση και είναι πολύ επικίνδυνη γιατί προδιαθέτει για οξέα καρδιαγγειακά συμβάματα. Μέχρι σήμερα δεν υπάρχει κάποιο ειδικό φάρμακο που θα μπορεί να καθυστερήσει αυτή τη διαδικασία. Η βιταμίνη Κ2 είναι μια φυσική, λιποδιαλυτή βιταμίνη που υπάρχει στο σώμα μας. Υπάρχει ως συμπλήρωμα διατροφής που αγοράζεται πάνω από τον πάγκο, χωρίς συνταγή γιατρού από φαρμακεία ή και σούπερ μάρκετ από εκατομμύρια ανθρώπων ανά τον κόσμο. Η βιταμίνη Κ2 δεν έχει παρενέργειες και δεν έχει προκαλέσει ποτέ τοξικότητα. Δεν είναι φάρμακο αλλά φυσικό συμπλήρωμα και είναι απόλυτα ασφαλές για ασθενείς υπό Περιτοναϊκή Κάθαρση. Έχει βρεθεί πως η βιταμίνη Κ2 μπορεί να απομακρύνει το ασβέστιο από τις αρτηρίες και με αυτόν τον τρόπο να βελτιώνει την υγεία των αρτηριών και να σας προστατεύσει από την εμφάνιση καρδιαγγειακών επεισοδίων. Αιμοκαθαιρόμενοι ασθενείς υπό Τεχνητό Νεφρό που λαμβάνουν βιταμίνη Κ2 δεν έχουν εμφανίσει σοβαρές παρενέργειές ή τοξικότητα ενώ αντιθέτως, βελτιώθηκε η κατάσταση των αγγείων τους και ζουν περισσότερο. Ο λόγος που διεξάγουμε αυτήν την έρευνα είναι για να διαπιστώσουμε αν αυτό το συμπλήρωμα διατροφής μπορεί να βελτιώσει την επασβέστωση των αγγείων σε ασθενείς υπό Περιτοναϊκή Κάθαρση.

**Είδος ερευνητικής παρέμβασης**

Αυτή η μελέτη θα περιλαμβάνει καθημερινή χορήγηση από του στόματος 1 χαπιού για 1,5 έτος. Στην αρχή της μελέτης θα μετρήσουμε με ένα υπέρηχο και ένα φορητό πιεσόμετρο την αγγειακή επασβέστωση και την 24ωρη αρτηριακή πίεση σας. Οι εξετάσεις αυτές θα επαναληφθούν στο τέλος της μελέτης για σύγκριση. Κάθε μήνα στην τακτική σας επίσκεψη στη Μονάδα Περιτοναϊκής Κάθαρσης θα συζητάμε όποιες ανησυχίες ή απορίες έχετε.

**Επιλογή συμμετεχόντων**

Προσκαλούμε όλους του ασθενείς υπό Περιτοναϊκή Κάθαρση που παρακολουθούνται στη Μονάδα μας να συμμετέχουν σε αυτή την μελέτη σχετικά με ένα νέο συμπλήρωμα που πιθανά προστατεύει από την επασβέστωση των αγγείων.

**Εθελοντική Συμμετοχή**

Η συμμετοχή σας στη μελέτη είναι εντελώς εθελοντική. Είναι απόλυτα προσωπική σας επιλογή αν επιθυμείτε να συμμετέχετε ή όχι και σε περίπτωση που δε θελήσετε, όλες οι ιατρικές υπηρεσίες και περίθαλψη που λαμβάνετε έως τώρα στη Μονάδα Περιτοναϊκής Κάθαρσης θα συνεχιστούν κανονικά και τίποτα δε θα αλλάξει. Ένα επιλέξετε να συμμετέχετε, έχετε το δικαίωμα να αλλάξετε γνώμη στην πορεία και να σταματήσετε τη συμμετοχή σας ανά πάσα στιγμή.

**Πληροφορίες για το συμπλήρωμα διατροφής βιταμίνης Κ2**

Το συμπλήρωμα που θα λάβατε είναι η βιταμίνη Κ2, που είναι μια φυσική βιταμίνη που ήδη υπάρχεις το σώμα σας. Εκατομμύρια άνθρωποι σε όλο τον κόσμο λαμβάνουν καθημερινά το συμπλήρωμα αυτό, καθώς έχει σχετιστεί με πολλές ευεργετικές δράσεις. Σκοπός της μελέτης είναι να εξετάσουμε αν η καθημερινή χορήγηση του συμπληρώματος βιταμίνης Κ2 προστατεύει την υγεία των αρτηριών των ασθενών υπό Περιτοναϊκή Κάθαρση. Η χρήση της βιταμίνης Κ2 δε συνοδεύεται από παρενέργειες εκτός από ελάχιστες φορές που μπορεί να προκαλέσει ναυτία ή διάρροια. Δεν υπάρχουν όμως άλλες παρενέργειές ή κίνδυνοι.

**Διαδικασίες και Πρωτόκολλο**

Για να ελέγξουμε αν η βιταμίνη Κ2 προστατεύει τα αγγεία σας, θα χρειαστεί να συγκρίνουμε τη δράση της με ένα εικονικό χάπι (placebo). Για να γίνει αυτό θα χωριστούν οι συμμετέχοντες στη μελέτη σε 2 ομάδες, σε μια ομάδα θα είναι εκείνοι που θα λαμβάνουν κάθε μέρα με το φαγητό ένα χάπι βιταμίνης Κ2 και στην άλλη θα λαμβάνουν το εικονικό χάπι. Το εικονικό χάπι είναι ένα χάπι που εξωτερικά θα μοιάζει ακριβώς με το συμπλήρωμα βιταμίνης Κ2, αλλά θα περιέχει μόνο ζάχαρη και νερό. Η επιλογή στις 2 ομάδες θα γίνει απολύτως τυχαία, σαν να πετάς ένα κέρμα. Κατά τη διάρκεια της μελέτης, κάθε μήνα θα συνεχίσετε να έρχεστε στα τακτικά ραντεβού σας στη Μονάδα Περιτοναϊκής Κάθαρσης, όπως πριν. Ένα σε αυτή την περίοδο έχετε οποιαδήποτε σκέψη, απορία ή ανησυχία, μη διστάσετε να τα συζητήσετε με εμένα ή οποιοδήποτε γιατρό της Μονάδας.

Στη αρχή και στο τέλος της μελέτης θα σας κάνουμε αιμοληψία και θα πάρουμε δείγμα από τα περιτοναϊκά υγρά σας για να εξετάσουμε την επάρκεια σας σε βιταμίνη Κ2. Επίσης θα μετρήσουμε την σκληρία των αρτηριών σας με μια απλή μέθοδο που περιλαμβάνει υπέρηχο και διαρκεί 10-15 λεπτά και θα σας ζητήσουμε να φορέσετε ένα φορητό πιεσόμετρο για 24 ώρες για να δούμε την αρτηριακή σας πίεση. Αυτή η μελέτη θα διαρκέσει 1,5 έτος. Εκτός από τις τακτικές μηνιαίες σας επισκέψεις στη Μονάδα, δεν απαιτούνται επιπλέον.

**Παρενέργειες και Κίνδυνοι**

Όπως προαναφέρθηκε η βιταμίνη Κ2 είναι ένα φυσικό συμπλήρωμα διατροφής και όχι φάρμακο και δεν έχουν αναφερθεί ποτέ παρενέργειες ή τοξικότητα σε ασθενείς με Χρόνια Νεφρική Ανεπάρκεια Τελικού Σταδίου, όπως είστε εσείς.

**Εμπιστευτικότητα**

Οι πληροφορίες και τα δεδομένα που θα συλλέξουμε από τους ασθενείς της μελέτης θα παραμείνουν εμπιστευτικά. Κανένας εκτός από τους υπευθύνους ιατρούς δεν θα έχει πρόσβαση στα δεδομένα σας. Οποιαδήποτε πληροφορία σχετικά με εσάς θα αναφέρεται στη μελέτη κωδικοποιημένη με αριθμούς (πχ δε θα φαίνεται στα έγγραφα «ο κ. Παπαδόπουλος» αλλά «ο ασθενής 543») και μόνο οι ιατροί-ερευνητές που συμμετέχουν στη μελέτη θα γνωρίζουν που αντιστοιχεί ο κωδικός.

**Κοινοποίηση των αποτελεσμάτων**

Μετά το τέλος της μελέτης, τα αποτελέσματα θα σας κοινοποιηθούν προτού δημοσιευτούν σε ιατρικά συνέδρια ή περιοδικά.

**Δικαίωμα Άρνησης ή Ανάκλησης**

Δε χρειάζεται να συμμετάσχετε στη μελέτη εάν δεν το επιθυμείτε. Εάν συμφωνείτε να συμμετέχετε, μπορείτε να αλλάξετε γνώμη και να σταματήσετε τη συμμετοχή σας ανά πάσα στιγμή το θελήσετε, είναι αποκλειστικά δική σας επιλογή και θα το σεβαστούμε.

**Με ποιόν να επικοινωνήσετε**

Εάν έχετε απορίες, μπορείτε να τις συζητήσετε τώρα ή αργότερα, ακόμα και μετά την έναρξη της μελέτης. Ανά πάσα στιγμή, μπορείτε να επικοινωνήσετε για τη μελέτη με τους ακόλουθούς ιατρούς:

Στέφανος Ρουμελιώτης, [st_roumeliotis@hotmail.com](mailto:st_roumeliotis@hotmail.com), 2313303855

Βασίλειος Λιακόπουλος, vliak@auth.gr, 2313303855

**Η παρούσα ερευνητική πρόταση έχει αναθεωρηθεί εγκριθεί από την Επιτροπή Βιοηθικής και Δεοντολογίας του Επιστημονικού Συμβουλίου της Ιατρικής Σχολής του Αριστοτελείου Πανεπιστημίου Θεσσαλονίκης. Αυτή η επιτροπή έχει ως καθήκον να διασφαλίσει ότι οι συμμετέχοντες στη μελέτη προστατεύονται από οποιαδήποτε βλάβη. Αν θέλετε να ενημερωθείτε περισσότερο για την Επιτροπή αυτή, επικοινωνήστε στα εξής στοιχεία: [Στ. Κυριακίδη 1, 54636, Θεσσαλονίκη, 2313303110, ahepahos@n3syzefxis.gov.gr].**

Μπορείτε να με ρωτήσετε ό,τι ερωτήσεις έχετε σχετικά με τη μελέτη. Έχετε καμία ερώτηση?

**ΜΕΡΟΣ II: Πιστοποιητικό συναίνεσης για συμμετοχή στη μελέτη**

Έχω διαβάσει τις παραπάνω πληροφορίες ή μου τις διάβασαν. Είχα την ευκαιρία να κάνω ερωτήσεις σχετικά με τη μελέτη και όποιες ερωτήσεις έκανα έχουν απαντηθεί ικανοποιητικά. Δίνω τη συγκατάθεση μου να συμμετέχω εθελοντικά στην ερευνητική αυτή μελέτη.

Ονοματεπώνυμο Συμμετέχοντος__________________

Υπογραφή Συμμετέχοντος ___________________

Ημερομηνία ___________________________

Ημέρα/Μήνας/Έτος

**Δήλωση του ερευνητή/ατόμου που παίρνει τη συγκατάθεση του ασθενή**

**Έχω διαβάσει με ακρίβεια το Δελτίο Πληροφοριών στον υποψήφιο συμμετέχοντα και στο μέτρο του δυνατόν βεβαιώθηκα ότι ο συμμετέχοντας καταλαβαίνει τις διαδικασίες και το πρωτόκολλο. Επιβεβαιώνω πως δόθηκε στο συμμετέχοντα η ευκαιρία να υποβάλλει ερωτήσεις σχετικά με τη μελέτη και ότι όλες οι ερωτήσεις που έθεσε ο συμμετέχων έχουν απαντηθεί ικανοποιητικά και σωστά. Επιβεβαιώνω πως ο συμμετέχων δεν έχει εξαναγκαστεί να δώσει τη παρούσα συναίνεση, αλλά την παρείχε ελεύθερα και εθελοντικά.**

**Ένα αντίγραφο της παρούσας** **Φόρμας ενημέρωσης και συγκατάθεσης για τη μελέτη έχει παρασχεθεί στο συμμετέχοντα.**

**Ονοματεπώνυμο Ερευνητή/ατόμου που παίρνει τη συγκατάθεση__________________**

**Υπογραφή Ερευνητή/ατόμου που παίρνει τη συγκατάθεση ___________________**

**Ημερομηνία ___________________________**

**Ημέρα/Μήνας/Έτος**
